# The Therapeutic Potential of the Endocannabinoid System in Age-Related Diseases

**DOI:** 10.3390/biomedicines10102492

**Published:** 2022-10-06

**Authors:** Ivona Maria Tudorancea, Mitică Ciorpac, Gabriela Dumitrița Stanciu, Cătălin Caratașu, Alina Săcărescu, Bogdan Ignat, Alexandra Burlui, Elena Rezuș, Ioana Creangă, Teodora Alexa-Stratulat, Ionuț Tudorancea, Bogdan Ionel Tamba

**Affiliations:** 1 Advanced Research and Development Center for Experimental Medicine (CEMEX), “Grigore T. Popa” University of Medicine and Pharmacy, 16 Universității Street, 700115 Iași, Romania; 2 Department of Medical Specialties II, “Grigore T. Popa” University of Medicine and Pharmacy, 16 Universității, 700115 Iași, Romania; 3 Department of Neurology, Clinical Rehabilitation Hospital, 14 Pantelimon Halipa, 700661 Iași, Romania; 4 Department of Neurology, “Grigore T. Popa” University of Medicine and Pharmacy, 700115 Iași, Romania; 5 Department of Rheumatology and Rehabilitation, “Grigore T. Popa” University of Medicine and Pharmacy, 700115 Iași, Romania; 6 Clinical Rehabilitation Hospital, 700661 Iași, Romania; 7 Oncology Department, “Grigore T. Popa” University of Medicine and Pharmacy, 700115 Iași, Romania; 8 Oncology Department, Regional Institute of Oncology, 700483 Iași, Romania; 9 Department of Morpho-Functional Sciences II, Discipline of Physiology, “Grigore T. Popa” University of Medicine and Pharmacy, 700115 Iași, Romania; 10 Cardiology Clinic “St. Spiridon” County Clinical Emergency Hospital, 700111 Iași, Romania; 11 Department of Pharmacology, Clinical Pharmacology and Algesiology, “Grigore T. Popa” University of Medicine and Pharmacy, 16 Universității Street, 700115 Iași, Romania

**Keywords:** cannabinoids, aging, endocannabinoid system, cannabinoid receptors, age-related diseases

## Abstract

The endocannabinoid system (ECS) dynamically regulates many aspects of mammalian physiology. ECS has gained substantial interest since growing evidence suggests that it also plays a major role in several pathophysiological conditions due to its ability to modulate various underlying mechanisms. Furthermore, cannabinoids, as components of the cannabinoid system (CS), have proven beneficial effects such as anti-inflammatory, immunomodulatory, neuromodulatory, antioxidative, and cardioprotective effects. In this comprehensive review, we aimed to describe the complex interaction between CS and most common age-related diseases such as neuro-degenerative, oncological, skeletal, and cardiovascular disorders, together with the potential of various cannabinoids to ameliorate the progression of these disorders. Since chronic inflammation is postulated as the pillar of all the above-mentioned medical conditions, we also discuss in this paper the potential of CS to ameliorate aging-associated immune system dysregulation.

## 1. Introduction

It is well established that the endocannabinoid system (ECS) is involved in the modulation of various physiological processes such as memory, pain, cognition, temperature, mood, feeding, and pregnancy [1,2]. In the past decades, ECS has gained substantial interest since growing evidence suggests that ECS also plays a major role in several physiopathological conditions due to its ability to modulate various underlying mechanisms [3]. For example, a neuromodulatory effect induced by ECS was recently described in neurodegenerative disorders such as Alzheimer’s and Parkinson’s disease. Additionally, ECS has been shown to play a significant role in inflammation associated with these medical conditions [4,5,6].

The ECS is comprised of endocannabinoids, cannabinoid receptors, and the proteins that are involved in the transport, degradation, and synthesis of cannabinoids, such as diacylglycerol (DAG) lipase isozymes α and β, fatty acid amide hydrolase (FAAH), monoacylglycerol lipase (MAGL), and N-acylphosphatidylethanolamine-selective phospholipase D (NAPE-PLD) [3,7]. Recent studies focusing on the modulation of the ECS have demonstrated that multiple signaling pathways are involved in its modulation. This particular feature is proven by the multifunctionality of its components [7]. It is well known that cannabinoids interact mainly, but without excluding other classes, with three classes of receptors: (1) G-Coupled Protein Receptors (GPCRs)—Cannabinoid receptors 1 and 2 (rCB1 and rCB2), (2) Ligand-sensitive ion channels (e.g., Transient Receptor Potential Vanilloid 1—TRPV1), and (3) nuclear receptors (e.g., nuclear receptor peroxisome proliferator-activated receptor gamma (PPAR-γ)) [3]. CB1 receptor was initially discovered and characterized in rat brain and thereafter in the human central nervous system [8,9]. Unlike rCB1, the second cannabinoid receptor (CB2) was initially cloned and characterized in two type of cells: in a human promyelocytic leukemic cell line and in rodent spleen cells [10]. In the past years, the main structural features of cannabinoid receptors CB1 and CB2 were clarified by using X-ray crystallography [11,12]. These modern methods paved the way for the synthetization of agonists and/or antagonists with a high selectivity for cannabinoid receptors leading thus to a complete description of various underlying rCB-related pathways [13]. The most important modulators of cannabinoid receptors, their mechanism of action, and the main findings in various experimental models are summarized in Table 1. 

The cannabinoid receptors 1 and 2 are found in a variety of tissues and organs and are the pillar of the ECS signaling complexity. Both rCB1 and rCB2 are G protein coupled receptors but they are characterized by structural differences and thus, the affinity for various endogenous or exogenous ligands vary accordingly. rCB1 is mainly found in the central nervous system into axons and presynaptic terminals in the amygdala and cortex, in GABAergic interneurons in the hippocampus, glial cells (astrocytes, oligodendrocytes), and microglia [37,38,39]. Interestingly, rCB1 are also involved in neurotransmission since they were found in glutamatergic, cholinergic, dopaminergic, and serotonergic systems. Remarkably, significant differences in receptor density were reported in these regions depending on physiological or pathological conditions [40]. rCB2 are found in different populations of circulating immune cells and cellular elements in the spleen and thymus and are mainly involved in the immune reactions [41,42]. Moreover, recent evidence advocates that rCB2 play a pivotal role in the reduction of progression of neurodegenerative disorders such as multiple sclerosis, Alzheimer’s, and Parkinson’s disease [43,44,45].

All the compounds which have either a natural or synthetic source, as well as an endogenous origin and are able to interact with abovementioned cannabinoid receptors are generally known as cannabinoids. The recent advances in the field of spectrometric methods made possible the isolation and extraction of Δ9-tetrahydrocannabinol (Δ9-THC) and cannabidiol (CBD)—the 2 main phytocannabinoids found in highest concentration in the *Cannabis sativa* [40,46]. Given the vast history of empirical use of *Cannabis* sp. due to its analgesic, anti-inflammatory, and anxiolytic properties, experimental studies have focused on identifying new potential therapeutic effects of Δ9-THC and CBD. For example, in animal models of multiple sclerosis, CBD has been shown to have a significant anti-inflammatory effect since it was able to decrease the concentration of proinflammatory cytokines interleukin 6 (IL-6), interleukin 12 (IL-12), tumor necrosis factor α (TNF-α), and interleukin 1 (IL-1) [47]. Moreover, recent evidence has shown additional benefits such as analgesic effects in rheumatoid arthritis, fibromyalgia, and oncological conditions [40].

The extensive research on phytocannabinoids have led to the discovery of two endocannabinoids: anandamide or N-arachidonoyl ethanolamine (AEA) and 2-arachidonoylglycerol (2-AG) [48]. Together with the enzymes involved in their metabolism such as FAAH, monoacylglycerol lipase, and NAPE-PLD, they represent the basis of the well-known ECS [49]. AEA and 2-AG represent the main signaling lipids for cannabinoid receptors and share many features such as structural similarity and synthesis mechanism. Interestingly, both of them are synthesized “on demand” as a result of increased intracellular Ca^2+^ concentration. The enzymes involved in the synthesis and degradation of AEA and 2-AG are sensitive to both intra- and extracellular calcium ions [50]. The biosynthesis of AEA is carried out under the action of NAPE-PLD, from the precursor N-arachidonoylphosphatidylethanolamine (NAPE) [51]. The enzyme involved in AEA degradation is FAAH which transforms AEA into arachidonic acid and ethanolamine. Some authors suggest the possibility that FAAH also metabolize 2-AG [52,53]. 2-Arachidonoylglycerol is generated into a two-step synthesis pathway by removal of inositol triphosphate (IP3) from arachidonoyl-containing phosphatidyl inositol biphosphate (PIP2), followed by removal of the acyl group at position 1 by a diacylglycerol (DAG) lipase [54]. 2-AG degradation is primarily carried out by MAGL and secondary by serine hydrolase α-β-hydrolase domain 6 (ABHD6) [7,55].

Endocannabinoids are implicated in various physiological and pathological processes. Changes in endocannabinoids concentration, as well as dysregulation of the ECS have been associated with pathological conditions, including, but not limited to: cancer, osteoporosis, neuromotor, neuropsychological, and neurodegenerative diseases, respiratory diseases such as asthma, cardiovascular diseases such as stroke, atherosclerosis, myocardial infarction, metabolic disorders, arrhythmias, and hypertension [3,56].

Synthetic cannabinoids (SC) were synthetized to overcome the undesirable effects of phytocannabinoids by optimizing and/or improving the pharmacological profile. For example, current research is focusing on obtaining new classes of analgesics based on SC. As we have previously concluded, replacing opioids with SC for pain management will present not only mechanistic benefits, but it will also reduce health costs and deaths associated with excessive opioid use [57].

Aging is a complex physiological process which is under the influence of different genetic and biological factors and is generally characterized by a progressive loss of functional integrity. Epidemiological studies predict that 1 out of 6 individuals will be over the age of 65 in 2050 and the number of the elderlies is likely to double in the next 3 decades with the biggest increase among the population of east and southeast Asia. In these regions, the number of people is expected to increase from 261 million to >570 million in 2050. Furthermore, the least developed countries will host an average of about 2/3 of the world’s population [58]. It is well known that population aging will significantly increase the incidence and prevalence of the disorders that associate an increased mortality and morbidity. Neurodegenerative and cardiovascular diseases, cancer, and diabetes are the most common aging-associated disorders which significantly increase the risk of death [59,60]. At the same time, aging is one of the most significant risk factors for the aforementioned pathologies.

Growing evidence suggests that CS may represent a promising therapeutic approach for these disorders. Moreover, all the encouraging results from experimental studies focusing on the role of both classical and newly described cannabinoid receptors pave the way for the use of cannabinoids in a variety of medical conditions. The most representative clinical studies focusing on cannabinoids effect in various age related-diseases are summarized in Table 2.

**Figure 1:**
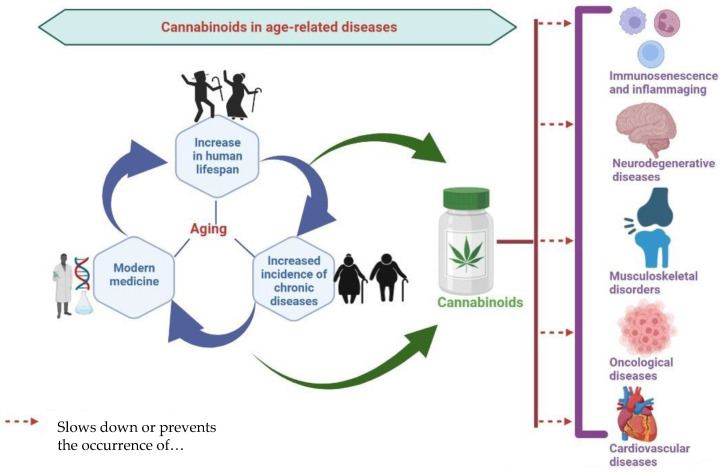
Schematic representation of the potential positive influence of cannabinoids use in various aging-related diseases.

However, to narrow the scope of the present paper we aimed to describe only the interaction between CS and most common age-related diseases such as neurodegenerative, oncological, skeletal, and cardiovascular disorders, together with the potential of various cannabinoids to ameliorate the progression of these disorders, as summarized in Figure 1. Since chronic inflammation is postulated as the pillar of all the above-mentioned conditions, we will also discuss in this paper the potential of CS to ameliorate aging-associated immune system dysregulation.

### The Physiology of Aging

The remarkable achievements of modern civilization in terms of living conditions together with the development of modern medicine have generated a considerable increase in humans’ lifespan. It is predicted that the abolition of pathological factors that are responsible for the main causes of death will increase the life expectancy from 50 to about 95 years [67]. It is clear now that the population aging will lead to a significant increase of the incidence and prevalence of chronic diseases that associate the highest mortality and morbidity rates. Among them, ischemic cerebro-cardiovascular diseases, neurodegenerative, oncological, and respiratory pathologies associate a continuously rising mortality rate and also have the biggest impact on overall prognosis and survival rate [68]. Thus, the abovementioned diseases clearly represent a challenge for the research community to improve the interrelation between chronic diseases and the increase in lifespan and aging.

Aging involves degenerative processes and functional changes at molecular, cellular, tissue, and whole body levels [69,70]. Understanding both the physiological and pathophysiological alterations that contribute to aging will provide a solid basis not only for the treatment, but also for the development of novel biomarkers which may reflect the risk of developing these age-related diseases.

The etiology of aging is complex and multifactorial. Experimental and clinical studies have failed to reveal until now the main mechanisms of aging and a plethora of theories have emerged in the past years [71]. We will discuss the most postulated theories of aging together with the potential of the CS to ameliorate various underlying mechanisms of the progression of aging-related diseases. One of the hypotheses proposed as the basic mechanism of aging is known as the telomeres theory of aging [72]. Telomeres have specialized deoxyribonucleic acid (DNA) protein structures that are found at the ends of all mammalian chromosomes and play a major role in covering the ends of the chromosomes to reduce DNA loss during cell replication. Telomere shortening may cause end-to-end fusion and chromosomal instability. The shortening phenomenon of telomeres to a critically short length during cell division has been associated with the finite replication capacity of somatic cells up to a certain critical point named the Hayflick limit, which precedes cellular senescence and apoptosis. Interestingly, telomere length decreases with age in somatic cells leading thus to the loss of the cell ability to divide [73]. On the other hand, this mechanism represents also a physiological response of the organism that prevents genomic instability due to the accumulation of damaged DNA [73,74,75,76,77]. Taken together, the length of the telomers may be considered as a key biomarker of the onset of senescence in human cell [78]. However, the telomere-dependent replicative senescence mechanism may differ between species and cell types leading to multiple limitations in translating the results from animals to humans [79,80]. Among the phenomenon of progressive shortening of telomers, other factors such as telomere dysfunction, genetic mutations, radiation and the presence of alkylating agents lead to the development and accumulation of senescent cells at different organism’s levels [81].

Emerging evidence indicates that senescent cells are characterized by a state of irreversible cell-cycle arrest together with a series of progressive and phenotypically diverse cellular states acquired after the initial growth arrest [82]. In addition, senescent cells release proinflammatory cytokines, chemokines, growth factors and proteases. This phenotype is known as the senescence-associated secretory phenotype (SASP) which generates both positive and negative effects [69]. For example, SASP may be effective in liver fibrosis and wound healing by interfering with remodeling processes of fibrous tissue. On the other hand, SASP may stimulate tumorigenesis by promoting angiogenesis or tumor growth [70,77,82]. Additionally, due to its paracrine mechanism of action, SASP may negatively impact adjacent cells by locally promoting a chronic state of inflammation, i.e., inflammaging, which is closely linked to several aging associated diseases such as rheumatoid arthritis, cancer, atherosclerosis, and neurodegenerative diseases [83,84]. Recent studies have shown that senescent cells may also induce a progressive functional deterioration in the tissues in which they accumulate contributing thus to the development of several age-related pathologies such as osteoarthritis, fibrosis, Alzheimer’s disease, pancreatitis, metabolic disorders, and atherosclerosis [70,71,77,83].

Additionally, cellular senescence is induced by high levels of reactive oxygen and nitrogen species (RONS) that further influence various components of SASP. The accumulation of altered macromolecules induced by RONS during oxidative processes that trigger the initiation of cellular senescence led to the elaboration of another theory of aging called the oxidative stress theory [84,85]. Although reactive oxygen species (ROS) are the products of endogenous aerobic metabolism, RONS result from both endogenous (nicotinamide adenine dinucleotide phosphate oxidase, myeloperoxidase, lipoxygenase and angiotensin II) and exogenous sources (pollution, tobacco, alcohol, heavy metals) [86,87,88]. As in the case of ROS, RONS resulting from cellular metabolism due to the alteration of the balance between their generation and removal may aggravate the oxidative stress [84,85].

Experimental and observational studies have shown that an increase in both ROS and RONS levels is associated with various age-related diseases. For example, it is clear now that physiopathological alterations of the cardiovascular system may also occur due to a decreased tolerance to reactive oxygen species. Moreover, the aged organism carries a high risk of atherogenicity due to a significant increase in oxidized low-density lipoprotein (LDL) levels [89,90]. ROS also modulate the activation of transcription factors that further influence the release of proinflammatory biomolecules. Moreover, the transcription of nuclear factor kappa-light-chain-enhancer of activated B cells (NF-kB) activated by TNF-α and RONS stimulates the genes involved in cell proliferation and carcinogenesis [84,91]. Thus, oxidative stress may activate immune cells and further exacerbate inflammation [84,92]. Recent studies have also shown that oxidative stress has detrimental effects on the normal function of neuronal cells [93]. The presence of active redox metals such as copper and iron, together with high concentrations of polyunsaturated fatty acids (PUFA) that are prone to lipid peroxidation, as well as low levels of antioxidant glutathione (GSH) may represent the main mechanisms involved in the development of neurodegenerative diseases such as Alzheimer’s, Parkinson’s, and Huntington’s disease [86].

Taken together, the telomers shortening, cellular senescence, and oxidative stress may represent the main mechanisms responsible for the development of aging-related diseases. Moreover, all these mechanisms are also capable of inducing various functional alterations of the immune system which further promote and maintain a prolonged status of chronic inflammation that aggravates the progression of aging-associated diseases.

## 2. Chronic Inflammation and Immunosenescence in Aging

It is now generally accepted that ECS dynamically regulates many aspects of mammalian physiology [94]. Recent evidence indicates that ECS may play a pivotal role in aging-related diseases such as neurodegenerative, inflammatory, and immune-related diseases, neoplasia, and cardiovascular disorders [95,96]. In contrast, the relationship between ECS, inflammatory and immune-related diseases, and aging is still under debate. Recently, a beneficial influence of the cannabinoids on the abovementioned relationship was described and ongoing studies are focusing on the hypothesis that cannabinoids may improve the pathophysiological mechanisms associated with inflammation and immune system dysfunction [97]. In the next paragraphs, we focused on the description of the ECS alterations and the therapeutic potential of the natural, synthetic and endogenous cannabinoids in all the before mentioned disorders.

### 2.1. Cannabinoids in Chronic Inflammation

Chronic inflammation and subsequent alterations of the immune system that occur with advancing age, i.e., immunosenescence, are strongly related [98,99]. The majority of the studies focusing on the concept of immunosenescence points toward the conclusion that this represents the leading cause of most biological changes related to aging and associated pathologies. Although many features of immunosenescence may be considered harmful, there are also adaptive or remodeling characteristics which are necessary to ensure survival and longevity, as long as they are kept within normal limits [98]. We will further describe the harmful consequences of the immunosenescence-induced inflammation and also the potential beneficial role of cannabinoid use to improve this relationship.

As mentioned above, aging involves a nonspecific state of chronic inflammation, a particular condition that is usually characterized by increased levels of multiple inflammatory biomarkers such as C-reactive protein (CRP), TNF-α, IL-6, and interleukin 8 (IL-8) [100,101]. Experimental studies have shown that cannabinoids may have anti-inflammatory effects since their administration in various age-related diseases led to positive outcomes on various subpopulations of inflammatory cells. For example, in an in vivo experiment, Kaplan et collaborators have demonstrated in an animal model of inflammation that CBD has a significant anti-inflammatory effect since it was able to decrease the circulating levels of IL-6 [102]. In another in vitro experimental study focusing on allergic contact dermatitis, CBD was shown to suppress the inflammatory state by the inhibition of synthesis of proinflammatory cytokines such as IL-6, IL-8, and TNF-α [103]. Furthermore, in an animal model of myocardial ischemia, CBD demonstrated notable cardioprotective effects due to its ability to significantly reduce IL-6 levels [104]. In all these experimental studies, the anti-inflammatory effects of CBD were mediated through the cannabinoid receptors CB1 and CB2 [102]. In addition, activation of G protein coupled receptor 55 (GPR55), inhibition of FAAH, activation of TRPV1 receptors, and gamma receptor activated by PPAR-γ peroxisome proliferator, as well as CB2/5HT1A heterodimerization represents additional pathways through which CBD exerts its anti-inflammatory effects [105,106].

Another component of the cannabinoid system is Δ9-THC which also showed various anti-inflammatory effects in both in vivo and in vitro studies. Interestingly, unlike CBD, Δ9-THC exerts its anti-inflammatory action by inhibiting T-helper (Th)-1 lymphocytes and promoting the activity of Th-2 lymphocytes [106]. Moreover, THC plays an important role in modulating the inflammatory response by suppressing proliferation, chemotaxis, phagocytosis and cytokine production. In a study on human monocytes and astrocytes, Δ9-THC was shown to suppresses inflammatory responses through the suppression of the IL-1b monocyte synthesis and subsequent IL-6 astrocytes production [107]. Thus, like CBD, Δ9-THC is also able to reduce the pro-inflammatory state mediated by IL-6. This is of paramount importance given that the majority of experimental and clinical studies have shown that an increased concentration of IL-6 is strongly correlated with high morbidity and mortality in the elderly [108]. Moreover, the inflammatory pathways encompassing pro-inflammatory cytokines such as IL-6, IL-8, and IL-1α have also been shown to play a critical role in the expression of cardiovascular risk factors such as hypertension, atherosclerosis, diabetes, and obesity which increases the risk for cardiovascular disease with age [84,109]. Taken together, these experimental results suggest that the modulation of pro-inflammatory cytokines such as IL-6 by ECS may have beneficial effects on aging-related diseases.

Experimental studies have shown that ECS is also involved in the modulation of TNF-α levels, a cytokine which is known to play a key role in the proinflammatory status associated with aging [101,110]. Interestingly, in an in vitro study on the wild murine glial cells, abnormal cannabidiol (an atypical synthetic cannabinoid) developed anti-inflammatory effects by reducing lipopolysaccharide (LPS)-induced TNF-α production. Furthermore, abnormal cannabidiol significantly attenuated lipopolysaccharide-induced TNF-α expression in astrocytes [111]. In addition, in a murine model of chronic liver failure due to excessive alcohol consumption, CBD suppressed the expression of several proinflammatory cytokines including TNF-α [112]. In another murine study on a model of anti-CD40 colitis, THC was also able to reduce the circulating levels of TNF-α, interleukin 17A (IL-17A), interleukin 22 (IL-22), and interferon-γ (IFN-γ), leading to a significant reduction in both systemic and local inflammation [113]. Granja et al. have shown in an in vitro study on murine neuronal cultures that a synthetic cannabinoid (VCE-003) is able to significantly reduce the secretion of pro-inflammatory cytokines such as TNF-α, IL-6, and IL-1β. Interestingly, the mechanism of action is mediated by the activation of PPAR-γ receptors and the anti-inflammatory effect may be enhanced by various phytocannabinoids with high affinity for this receptors [114]. Taken together, the abovementioned results suggest that the inhibition of pro-inflammatory pathways through ECS may be a promising approach to ameliorate various aging-related pathologies and to improve thus the prognostic and quality of life of these patients.

It Is well known that the inflammation plays a key role not only in the onset, but also in the progression of age-related diseases. CRP is an inflammatory marker which has predictive roles in morbidity and mortality in the elderlies since its increased circulating level is usually associated with the development and progression of various cardiovascular, metabolic and neurodegenerative diseases [100,115,116]. Although the cannabinoids induced anti-inflammatory effects have also been studied in the CRP-mediated inflammatory pathways, their role still remains controversial. For example, the results from a study on 1115 recently active cannabis smokers and 8041 non-smokers showed that recent and active cannabis smokers have lower serum CRP levels when compared to non-smokers [117]. Although this effect may be explained by gender differences, body mass index (BMI) disparities, and anti-inflammatory medication use, a retrospective analysis from Population Assessment of Tobacco Health (PATH) study found that recent cannabis use is associated with reduced levels of systemic inflammation biomarkers, including CRP [115,118]. Taken together, the abovementioned results indicate that cannabinoids present anti-inflammatory effects that may have additional benefits to classical drugs in reducing the progression of chronic inflammation associated with aging.

### 2.2. Immunosenescence and Cannabinoid System

It is clear now that a pro-inflammatory state is a hallmark of aging. The elderly population is characterized by an inability to develop a proper anti-inflammatory response to various exogenous and/or endogenous aggressors due to a particular state of immunosenescence [101]. Immunosenescence emerges due to a reduction in both qualitative and quantitative response of naive peripheral T and B lymphocytes as we age. Since elderly individuals have a reduced ability to respond to non-self structures and an increased susceptibility to infections, they have a higher probability to develop a chronic inflammatory state which further promote the development of various age-related diseases [119]. For example, longitudinal studies have shown that severe reduction in B cell count, reversal of CD4/CD8 T cell ratio, and reduced proliferative response lead to a decreased survival rates among the octogenarians and nonagenarians [120]. Recent studies in human and rodents have shown that both T and B lymphocytes express cannabinoid receptors on the surface of their membranes [121]. Although CBD demonstrated immunosuppressive and immunomodulatory effects in some experimental studies, there are still controversies among authors regarding the ability of this cannabinoid to reduce the pro-inflammatory status. Methodological conditions such as the concentration of the CBD used, cell culture parameters, the presence or the absence of the serum, and the modality of the immune system stimulation may explain in part these differences [102]. Although many of these conflicting results may be explained in part by the differences of methodological approaches, there is a continuous need for further studies focusing on the involvement of ECS on inflammatory state in general and on immunosenescence in particular [121].

The influence of cannabinoids on B lymphocytes has also been extensively studied. For example, in a study on mice investigating the role of 2-AG on B lymphocytes, the authors showed that this cannabinoid is able to induce the migration of B220 + CD19 + B cells through the chemoattraction of I B cells mediated by CB2 receptors [122]. 2-AG appears to modulate the differentiation of B cell populations and promote their migration during the development of the immune response [121].

The changes that occur with age influence both the adaptive and innate immune cells. For example, an increase in the number of Natural Killer (NK) cells in healthy aging has been reported as physiological. NK cells are considered the main subgroup of lymphocytes involved not only in antimicrobial defense, but also in removal of senescent cells, modulation of inflammation, and initiation of the adaptive immune response [119,123]. Additionally, both NK cell subpopulations express rCB1 and rCB2 and release considerable amounts of endocannabinoids [121]. Interestingly, the ECS is also involved in the modulation of the activity of this cell sub-population. In a study of CB^−/−^ deficient mice, the authors showed that endocannabinoids with affinity for rCB2 play a key role in suppressing cytokine production by lung NK cells. Moreover, these endocannabinoids were able to decrease the inflammation associated with allergic asthma [124].

Other components of the immune system that undergo functional changes with aging are neutrophils and macrophages. It is known that the alteration of the functions of these cells will alter the phagocytic response to a bacterial invasion. Moreover, macrophages are characterized by a reduced chemotaxis in the elderly [119]. Interestingly, both macrophages and neutrophils express rCB1 and rCB2, and the activation of CB2 receptors in neutrophils reduce the release of metalloproteases which reduce the vulnerability of atherosclerotic plaque [56,125]. In addition, in vivo studies have shown that 2-AG is a potent regulator of the host defense and is involved in activating human neutrophils by stimulating the release of kinases, myeloperoxidases, B4 leukotrienes, and by mobilizing cellular calcium [121].

Macrophages are mainly involved in the clearance of apoptotic cells and pathogens and interactions with other immune cells. Studies on murine macrophages have shown that 2-AG inhibits TNF-α and IL-6 production and promotes the anti-inflammatory effects of macrophages. Conversely, in the same study, 2-AG increased inducible nitric oxide synthase-dependent nitric oxide production based on arachidonic acid pathway [121]. Moreover, studies involving cancer patients have shown that targeting cannabinoid receptors from macrophages membrane may be helpful in reducing disease progression [126].

It is clear now that the ECS is involved in the regulation of the immune system at many levels. The cannabinoid system–immunosenescence–aging relationship remains provocative. However, encouraging results from experimental studies support the use of cannabinoids in various immune disorders which are associated with various aging-related neurodegenerative, autoimmune, cardiovascular, and cancer diseases [56].

#### Changes of Cannabinoid System in Immune Dysregulation Associated with an Increased Risk of Infections in Elderly Patients

**Figure 2:**
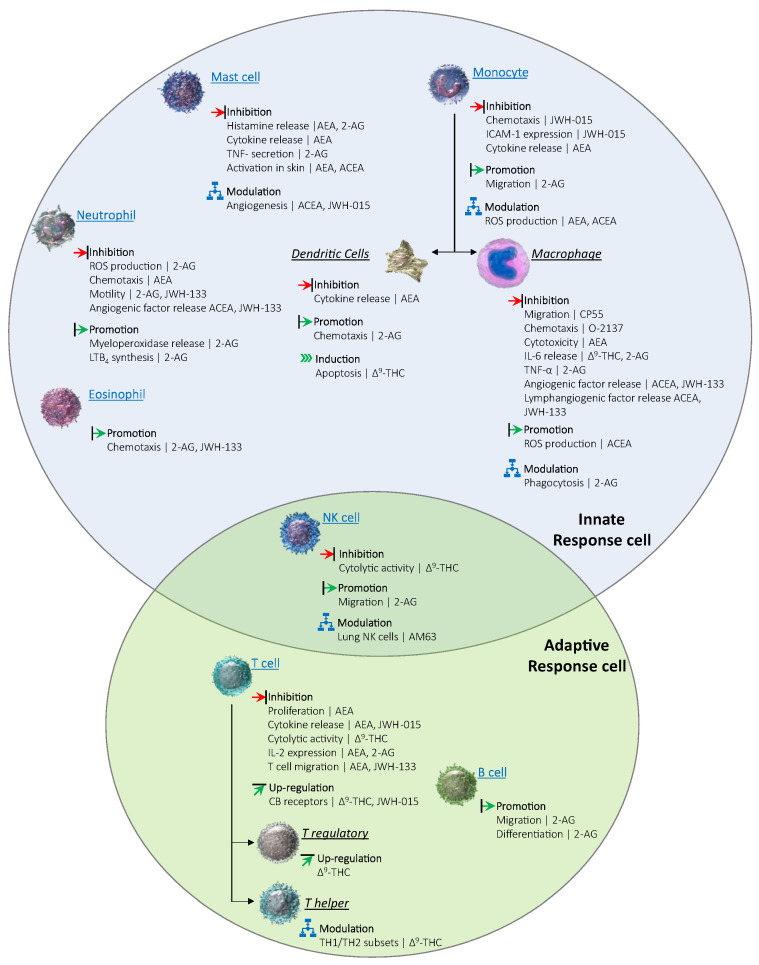
Schematic representation of main effects of the cannabinoid receptors on cells of innate and adaptive immune system;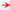 = inhibition of; 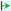 = promotion of; 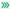 = induction of; 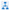 = modulation of.

Observational studies have reported that elderly patients present an overall higher risk of infection usually associated with an increased severity and poor prognosis. Immunosenescence, define as the state of dysregulated immune function that occurs as people age, is among of the main risk factors which increase the susceptibility of the elderly to infection [127]. It is well known that the immune system cells undergoes age-related changes through both intrinsic and extrinsic aging pathways leading to immunosenescence [128]. Dysregulated immune cell production and altered peripheral selection processes related to precursor cell’s differentiation are related to intrinsic aging pathways. In contrast, the immune cells’ lifelong exposure to antigenic and other stressors from the internal and external environment are usually related to extrinsic aging pathways [128]. Interestingly, recent evidence has reported that the ECS is involved in the modulation of the immune homeostasis as it may play the role of a gate-keeper for different immune cells [121,129,130,131]. As shown in Figure 2, all the immune cells also present cannabinoid receptors and perform various roles such as inhibition, modulation, or promotion of different inflammatory pathways [132]. For example, different immune cells interactions which contribute to the host defense mechanisms are also signaled through cannabinoid receptors [127]. Among a variety of alterations of physiological systems, aging is also linked to the immune system dysregulation which is known to be associated with an increased susceptibility to infectious diseases. Despite the increased number of studies focusing on CS, the knowledge regarding the age-related-changes of cannabinoid system and how immune dysregulation are linked to the ECS cannabinoid receptors in elderly patients is still fragmented, missing, or reduced to a few organs. Therefore, additional studies are required in order to achieve promising therapeutic benefits of the cannabinoids in aging therapy.

## 3. The Interplay between Cannabinoid System and Age-Related Diseases

### 3.1. Cannabinoids Potential in Age-Related Neurodegenerative Diseases: Alzheimer’s Disease

Alzheimer’s disease (AD) is a neurodegenerative condition characterized by a gradual cognitive decline and behavioral impairment that interfere with professional and social functioning [133]. According to the Alzheimer’s Association, the disease accounts for around 60 to 80% of dementia in elderly individuals, with advancing age being the strongest risk factor [134]. Currently, it is estimated that 50 million people worldwide suffer from AD and the prevalence is expected to double every two decades due to the increase of life expectancy and the rapidly aging population [134,135].

The continuous discovery of new signaling pathways in AD reflects the multifactorial pathophysiology of the disease, which is not yet fully understood [136]. Neuropathological hallmarks of AD include deposition of amyloid-β (Aβ) plaques and the presence of neurofibrillary tangles. These are strongly correlated with neuronal loss and neurodegeneration [137]. In the progression of the disease, it is believed that different physiological pathways play major roles, such as apolipoprotein E-mediated metabolism and cholesterol transportation, energy utilization, neuroinflammatory response, and vascular burden [138].

Despite the efforts made in the field of pharmacological research, acetylcholinesterase inhibitors remain the mainstay treatment option which provide only symptomatic relief without reducing the progression of the disease [133,139]. A plethora of factors contribute to the difficulty of developing effective therapies for AD such as gaps in knowledge regarding the molecular alterations and biological mechanisms in the brain that lead to the development of the disease. Furthermore, an inadequate number of patients enrolled into clinical trials and a prolonged time required to complete interventional studies represent additional factors which contribute to the inability to develop effective therapies [140]. For example, in the last 20 years, only Aducanumab, which represents a significant milestone for the treatment of AD, was approved for use in humans by the US Food and Drug Administration (FDA) in June 2021—on the condition of further successful trials [141]. The drug is an antibody that targets Aβ which decrease the number of Aβ plaques present in the brain and has the theoretical potential to ameliorate the cognitive deterioration typical for AD. Because the only target of aducanumab is Aβ plaques, the other features of the disease such as brain cell death or neuroinflammation remain unaddressed. Thus, the concept “one molecule–one target–one disease” fails to provide a comprehensive solution for AD treatment due to the complex and multilayered nature of the disease [142]. As a result, the ideal treatment for AD should be able to modulate the disease through multiple mechanisms rather than targeting a single dysregulated pathway. In this context, exogenous and endogenous cannabinoids are an attractive and promising target against this condition, supported by growing evidence of improved Alzheimer’s symptoms in various disease models after exposure to these compounds. This improvement may result from the modulation of the endocannabinoid system, as well from their positive effects on neuroinflammation, Aβ and tau processing, microglial activation, oxidative stress, mitochondrial dysfunction, glucose uptake in the brain, and excitotoxicity [143,144,145].

#### The Link between the Cannabinoid Systems and Alzheimer’s Disease through Its Effects on Inflammation, on the Immune System and on Oxidative Stress

**Figure 3:**
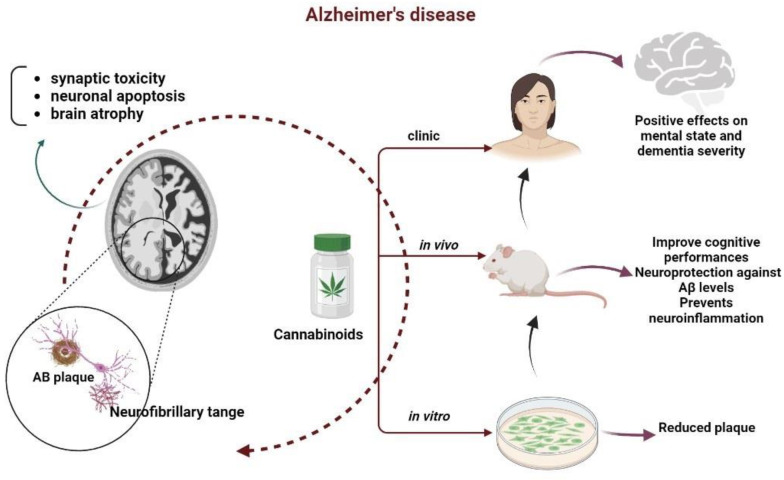
Schematic representation of the involvement of cannabinoids therapy in Alzheimer’s disease.

Early scientific evidence that cannabinoid compounds may be effective therapeutic tools for treating AD was obtained from the study of the ECS [44]. As mentioned before, the ECS contains at least two well-described receptors (rCB1 and rCB2). CB1 receptors are widely expressed in the central nervous system where they are involved in the regulation of the main functions of the brain [146]. In addition, CB1 receptors play a significant role in protecting against neurotoxicity and promoting repair mechanisms in response to neuronal injury [147,148]. Although CB2 receptors are mainly expressed in the immune system with relatively low expression in neuronal cells, specific CB2 receptor agonists have gained major attention in AD due to their lack of psychoactive properties [44,149]. Interestingly, it has been shown that CB2 receptors are selectively overexpressed in cells associated with Aβ enriched neuritic plaques in AD samples from postmortem human brains [150]. The growing interest in cannabinoids as a promising neuroprotective therapy in AD is based on their ability to decrease neuroinflammation through the activation of the CB1 and CB2 receptors. Moreover, they are also able to reduce the pathological action of Aβ and promote brain repair mechanisms [151,152,153,154]. In this regard, recent studies have shown that CB1 receptor activation maintains the viability of neurons by suppressing pro-apoptotic signaling pathways and by decreasing Aβ-mediated lysosomal membrane permeabilization [155,156]. CB2 receptors, expressed mainly in microglia, may suppress the AD neuroinflammatory processes by their immunomodulatory effect. Thus, in vitro studies have suggested that the release of pro-inflammatory cytokines in microglial cell cultures exposed to Aβ peptide can by reduced by selective agonists JWH-133, JWH-015, HU-308, and CB1 + CB2 agonists HU-210 and WIN55,212-2 [151,157]. These results were confirmed in animal models of AD, in which chronic administration of these compounds resulted in reduced microglial reactivity and decreased levels of various proinflammatory cytokines [151,153]. Moreover, in transgenic mice models of AD an improved cognitive performance was obtained after CB1 and CB2 targeting, as summarized in Figure 3 [158,159].

Another significant effect of the CS in AD is on oxidative stress. Two studies have shown that CB2 agonists may decrease the production of free radical nitric oxide induced by exposure to Aβ in microglial cell culture [151,157]. However, these results could not be replicated in a glioma cell line [160]. A series of in vivo studies have also shown that activation of CB2 receptors decreases oxidative stress injury and stimulates anti-oxidative stress responses; long-term treatment with low-dose of JWH-133 reduces the expression of hydroxynonenal adducts and increases the levels of both superoxide dismutase 1 and 2 around plaques in APP/PS1 transgenic mice [159]. The underlying mechanisms by which CB2 receptors mediate these antioxidant effects are not completely elucidated. Although it has been suggested that the CB1–CB2 agonist THC improves mitochondrial function leading thus to a reduction in free radicals, additional studies are needed to support this hypothesis [161].

Cannabinoids can also modulate both peripheral and cerebral immune system by influencing the expression of cytokine and T cell subpopulations, ameliorating thus the balance between neurodegeneration and neuroinflammation [162]. Interestingly, immune cells may produce endocannabinoids and may be influenced by cannabinoid analogues [163]. The use of cell and animal models that reproduce some of the cerebral abnormalities that occur in AD has led to the discovery of the therapeutic potential of cannabinoids (Table 3). It is particularly noteworthy that the few clinical trials to date also support the use of these compounds to alleviate some of the behavioral alterations associated with AD.

Although a plethora of scientific advances on the functional relevance of cannabinoids in AD were achieved in the past decades, a series of outstanding research questions still remain. For example, the beneficial effects of cannabinoids in neurodegenerative diseases such as dementia is still controversial as reported by Krishnan et al. [175]. Thus, a more detailed knowledge of the mechanisms of action of these cannabinoid compounds on animal models of Alzheimer’s and other neurodegenerative diseases is required for a better optimization of future therapies.

### 3.2. Cannabinoids Potential in Age-Related Neurodegenerative Diseases: Parkinson’s Disease

Parkinson’s disease (PD) is a neurodegenerative disorder characterized by basal ganglia (BG) dysfunction due to the loss of dopaminergic neurons in substantia nigra pars compacta (SNc) [176]. Growing evidence supports the hypothesis that neuroinflammation and oxidative stress are the main contributors to the neurodegenerative processes in PD [177]. It has also been suggested that the alteration in α-synuclein proteostasis may be an epiphenomenon mediated by inflammation [178]. There are also theories regarding the ability of extracellular α-synuclein to activate glial cells and induce neuroinflammation [179]. Higher concentrations of proapoptotic proteins have been found in the BG and the cerebrospinal fluid of PD patients. Striatal activated microglia that synthetize proinflammatory cytokines, such as transforming growth factor-β (TGF-β), interleukin 1β (IL-1β), IL-6, IFN-γ, and IL-1, have been detected as well [180,181]. Activated glial cells also seem to produce ROS and RONS in the substantia nigra (SN) [182,183].

Several alterations of the ECS in PD have been described [45,184]. Whether these modifications are compensatory and meant to limit the effects of dopamine loss in the BG or whether they contribute to the development of the motor symptoms in PD is still a matter of extensive debate, but the use of cannabinoids as a therapeutic option for this condition has become of interest [45,185].

The cannabinoid receptors expressed in the BG are rCB1, rCB2, GPR55, and TRPV1. Their presence is indicative for their role in the modulation of various signaling pathways present at this level [186,187,188]. rCB1 is found in gamma-aminobutyric acid (GABA)ergic and glutamatergic neurons projecting to globus pallidus pars interna (GPi), globus pallidus pars externa (GPe) and substantia nigra pars reticulata (SNr). Even though this receptor has not been discovered in dopaminergic neurons, it seems to modulate dopamine release through the GABAergic and glutamatergic neurons nearby and the formation of heteromers with the D1 and D2 dopamine receptors [45,188,189]. rCB1 seems to have different effects depending on where it is expressed [188]. The activation of this receptor in the GPi and SNr inhibits glutamate release from the afferent neurons of the subthalamic nucleus and seems to alleviate the symptoms and signs of PD. Conversely, enhanced transmission in the GPe may exacerbate the disease by reducing GABA reuptake at this level [188,190,191]. rCB2 is expressed in the striatum, globus pallidus, dopaminergic neurons in the ventral tegmental area, SNr, and basal thalamus [192,193,194,195,196]. Several studies have demonstrated the presence of this receptor in the cytosol and axon terminals of nigrostriatal dopaminergic neurons. However, rCB2 is mainly found in astrocytes and microglia and involved in the regulation of neuroinflammation [197,198]. TRPV1 is expressed by glial cells, nigrostriatal dopaminergic neurons in the BG, and the tyrosine hydroxylase-positive neurons of SNc. Through their presence in dopaminergic neurons, TRPV1 and rCB2 seem to be directly involved in the regulation of dopamine release [188,199,200,201].

In idiopathic and experimental PD, the ECS seems to reorganize at the level of the BG. An increase in the activity of rCB1 and AEA levels and a decrease in cannabinoid clearance have been described [45]. Another important finding is that rCB2 expression is upregulated in 1-methyl-4-phenyl-1,2,3,6-tetrahydropyridine (MPTP) treated mice and this seems to be a part of a neuroprotective mechanism which prevents the activation of microglia, the expression of astroglial myeloperoxidase and the disruption of the blood-brain barrier [182,183].

Because of the modifications in the ECS that have been identified in PD patients and PD models and the involvement of the cannabinoid receptors in the regulation of circuits within the BG, rCB1 and rCB2 have become new potential therapeutic targets. Most rCB1 agonists that have been tested had an inhibitory effect on dopamine release in the BG and therefore they are not expected to show any improvement in PD patients [45]. However, rCB1 agonists may prove useful in the attenuation of tremor and L-3,4-dihydroxyphenylalanine (L-DOPA)-induced dyskinesia [202,203,204]. A study by Song et al. also revealed that the chronic use of WIN 55,212-2, a rCB1 agonist, reduced the abnormal behavioral changes caused by L-DOPA in rat models of 6-hydroxydopamine (6-OHDA)-induced PD [205]. WIN 55,212-2 had been shown to inhibit the accumulation of α-synuclein and parkin as well [206]. A study on rats with 6-OHDA-induced lesions compared the effects of L-DOPA with those of an rCB1 antagonist called rimonabant and demonstrated that both L-DOPA and rimonabant improved stepping and the combined administration of L-DOPA and rimonabant had a better effect than either drug alone [207]. Moghaddam et al. conducted a study using both an rCB1 antagonist, AM251, and an agonist, ACPA, and measured the catalepsy in PD models of reserpinized rats and normal controls. AM251 alleviated the catalepsy in a dose dependent manner. In contrast, the administration of ACPA increased the catalepsy [208]. Such studies prove that rCB1 antagonist could be the next step as drug of choice in the alleviation of motor symptoms in PD. Cannabinoids may also improve non-motor symptoms in PD. An experimental study on rodents has shown that the systemic administration of CBD led to an increase in total sleep time by increasing AEA levels and having a modulatory effect in regions expressing rCB1 and involved in the sleep–wake cycle [209,210].

In regard to rCB2, preclinical trials have shown that the activation of this receptor suppresses microglial activation, therefore reducing neuroinflammation [211]. JWH-015, an rCB2 agonist, had a protective effect against MPTP-induced degeneration in mice by reducing the activation of microglia by MPTP. The same study also demonstrated that there was an exacerbation in MPTP toxicity following the genetic ablation of rCB2 [183]. The fact that rCB2 stimulation is involved in neuroprotection was also suggested in a study on tetrahydrocannabivarin (Δ9-THCV). Both the acute and chronic administration of Δ9-THCV led to a preservation of tyrosine hydroxylase-positive neurons in the SN of rats with 6-OHDA-induced lesions [212]. In support of these findings, another study using the same animal model showed that rats treated with GW842166x, a selective rCB2 agonist, scored better in balance beam walking, pole, grip strength, rotarod, and amphetamine-induced rotation tests [213]. In rotenone-induced animal models of PD, there is clear evidence of oxidative stress, loss of antioxidant enzymes, and enhanced production of proinflammatory cytokines, such as IL-1β, IL-6, and TNF-α. Treatment with the selective rCB2 agonist β-caryophyllene reduced the levels of these cytokines, prevented glutathione depletion, decreased lipid peroxidation, and increased the concentration of antioxidant enzymes [214].

Data on the neuroprotective effect of Δ9-THC and CBD were also provided by several studies [31,215,216,217]. Δ9-THC provided direct neuroprotection in PD-induced SH-SY5Y cell cultures and marmoset models of PD which were treated with Δ9-THC had a major improvement in locomotion. It has been hypothesized that rCB1 stimulation by THC in the striatum is able to overrule the inhibitory effects upon movement determined by the activation of rCB1 in the GPe [218,219,220]. Regarding CBD, its administration led to a downregulation of glycogen synthase kinase-3, which seems to be a major inhibitor of the WNT/β-catenin pathway. The WNT/β-catenin pathway is a signaling system that amplifies oxidative stress and inflammation and has been shown to be part of the metabolic reprogramming that characterizes PD [216]. CBD also seems to upregulate Cu/Zn-superoxide dismutase and, therefore, increases the endogenous mechanisms of defense against oxidative stress [215]. Through a preferential action on the astrocytes and the stimulation of the TRPV1 receptor, CBD also enhances the endogenous neuroprotection provided by the ciliary neurotrophic factor and, thus, maintains the viability of dopaminergic neurons [31].

Given the growing evidence for the benefits of administering cannabinoids in PD animal models, interest for studying their effects in PD patients has risen [221]. A survey of 339 patients showed that 25% had taken cannabis and 45.9% of these reported some benefit in regard to general improvement, tremor, bradykinesia, rigidity, and L-DOPA induced dyskinesia [222]. The improvement of motor symptoms was also described in a smaller study on 22 patients. In addition, some patients reported an improvement in the quality of sleep and a decrease in the visual analog scale of pain score could also be identified [223]. Several other uncontrolled trials have found improvement in the motor symptoms and also non-motor symptoms, such as pain, mood, and sleep [224,225,226]. An open-label study focusing on CBD administration in 6 PD patients described lower Unified Parkinson’s Disease Rating Scale (UPDRS) score with CBD use. The psychotic symptoms were also significantly decreased in these patients [227].

Only a few randomized controlled trials (RCTs) testing the use of cannabinoids in PD have been conducted and they provided contradictory results [221]. Two RCTs, one studying the effects of rimonabant, and the other Cannador^®^, a plant extract with a Δ9-THC to CBD ratio of 2:1 and standardized Δ9-THC content, failed to show significant improvement of the UPDRS scores [220,228]. In contrast, a RCT evaluating the effects of nabilone, a rCB1 and rCB2 agonist, on the L-DOPA induced dyskinesia showed significant improvement at a total dose of 0.03 mg/kg body weight when half of the dose was administered 12 h before and the rest 1 h before an acute L-DOPA challenge [229]. A dose of 75 mg/day or 300 mg/day of CBD did not provide a statistically significant improvement in the UPDRS, but it increased the quality of life [210]. An acute dose of 300 mg of CBD also provided a significant anxiolytic effect and a decrease in tremor amplitude [230].

The involvement of the ECS in the regulation of movement and the complex alterations within this system that become apparent in PD are far from being deciphered. Preclinical data show promising results regarding the use of cannabinoids in PD. Nevertheless, the clinical data is very lacking. More evidence is needed before cannabinoids become a viable option in the treatment of PD.

### 3.3. Cannabinoids Potential in Age-Related Neurodegenerative Diseases: Multiple Sclerosis

Multiple sclerosis (MS) is a chronic inflammatory autoimmune disease which involves complex interactions and underlying mechanisms which are not yet fully understood. Although many clinical and experimental studies focus on discovering new treatment strategies to improve the symptoms, quality of life, and overall prognosis of MS, no significant progress was achieved in the past decade. Thus, the cannabinoid approach in MS has evolved as a promising alternative not only for the improvement of symptomatology, but also for their potential to increase the efficacy of existing drugs since recent clinical evidence has shown that cannabinoids may ameliorate the symptomatology of pain, fatigue, depression, tremor, or sleep disorders [231]. For example, approval of Nabiximols (Sativex^®^), a 1:1 mixture Δ9-THC and CBD for MS related spasticity was a real progress for the management of this disease. From the first studies of Collin et al., it was recognizable that the improvement was significantly higher in subjective patient-reported measures than in objective parameters [232]. A plethora of further observational and randomized studies have confirmed the efficacy of Nabiximols, cannabis, and other cannabinoids to control spasticity, pain, sfincterian problems, and sleep disturbances in MS patients [64,233,234,235,236,237,238].

In patients with an advanced stage of MS, pain is a common symptom and may have various forms such as neuropathic pain, headache, trigeminal neuralgia, joint and muscle pain due to motor deficits, and walk abnormalities or spasticity. The antalgic effect induced by cannabinoids may be partially explained by a central mechanism which involves the restoration of cortical pain gating mechanisms, most likely through the modulation of sensory–motor cortical integration [239]. In a systematic review published by Longoria et al., pain reduction was found at −3.42 points after cannabinoids administration when compared to a control group [240]. In another paper focusing on the effects of cannabinoids on MS neuropathic pain, Jones and collaborators identified a clinically relevant difference between placebo and treatment groups but without reaching statistical significance [241]. Although modest evidence for the use of cannabinoids in MS for alleviating pain was reported by Jones et al., the aforementioned results pave the way for further experimental studies focusing on finding promising cannabinoids compounds which may have a more pronounced antalgic effects.

Although case reports and nonrandomized trials found evidence that cannabinoids may provide some benefits in MS-related tremor, none of the placebo-controlled trials reviewed by Pourmohamaddi and collaborators found any significant differences [242]. In a recent survey, cannabis consumption in the past 3 months in MS patients was associated with an improvement of bladder symptoms such as urinary frequency, urinary urgency, bladder leakage and wetness, pad use, and bladder emptying [243]. The same results were reported in a systematic review focusing on efficacy and tolerability of cannabinoids in MS which showed that bladder symptoms are significantly improved by the use of cannabis or cannabinoids [43].

Data from experimental studies using animal models of MS show a favorable effect of cannabinoids on clinical and biological parameters. For example, significant improvement in disability and behavior was described in experimental autoimmune encephalomyelitis (EAE) after administration of cannabinoid oil extract formulations. These results were based on complex biological mechanisms and included the reduction of TNF-α production and the enhancement of brain-derived neurotrophic factor (BDNF) synthesis [244].

Cannabinoids may play an important role in modulating the complex physiopathology of MS and may be used as immune modulators, neuroprotectors, or remyelination promoters. Although current MS treatment approaches are focused on the modulation of the immune system by using substances such as cladribine, alemtuzumab, or even bone marrow transplant, significant results were not yet obtained. Recent evidence has suggested that endocannabinoid system dysregulation contributes to the progression of inflammatory and degenerative processes associated with MS. For example, in MS patients, the ECS’s components were found altered not only in cerebrospinal fluid (CSF), but also in plasma and peripheral lymphocytes in different patterns depending on MS type and severity class [245,246,247,248]. Moreover, the involvement of cannabinoid systems in various MS related pathological mechanisms was confirmed not only by pharmacological modulation of receptors and enzymes, but also by their genetic deletion [249]. Interestingly, both neuroprotective (usually obtained through the activation of CB1 receptors) and anti-inflammatory (usually linked to CB2 activation) cannabinoids-induced effects are abolished by specific receptor blockade. Additionally, experimental studies have shown that neuroprotection is at least in part explained by the modulation of glutamate release which is dependent on CB1 receptors [250,251].

Other pathways may also contribute to the neuroprotective and anti-inflammatory cannabinoids dependent effects. VCE-004.8, a derivative of CBD, has a dual PPAR-γ and CB2 agonist action and also activates the HIF pathway in oligodendrocytes and microglia cells. In EAE and Theiler’s virus-induced encephalopathy (TMEV), VCE-004.8 enhanced migration of oligodendrocytes, prevented demyelination, axonal damage, immune cells infiltration, and downregulated the expression of several genes associated with MS physiopathology [252]. Hydroxy CBD enantiomers (HU-446, Hu465) prevented myelin oligodendrocyte glycoprotein (MOG) stimulated T cells to produce interleukin 17 (IL-17) via a CB1/CB2 independent mechanism [253].

Autoimmunity was postulated as another mechanism involved in the physiopathology of MS. Briefly, autoimmunity in MS comprises persistent activation of local astrocytes, microglial cells, recurrent and persistent infiltration of both peripheral leukocytes, and soluble inflammatory mediators. The immune aggression involves mainly cells of adaptive immunity such as CD8 and CD4 Th1 and Th17 lymphocytes. B-cells function as APCs and also produce autoantibodies that have been shown to contribute to neurodegeneration and cortical demyelination, particularly in the case of meningeal ectopic B cell follicles [254]. Intriguingly, in Theiler’s murine encephalomyelitis virus-induced demyelinating disease (TMEV-IDD), activation of PPAR-γ nuclear receptors by a CB agonist (WIN55,212-2) led to downregulation of intercellular adhesion molecule-1 (ICAM-1) and vascular cell adhesion molecule-1 (VCAM-1) in brain endothelium. Moreover, a reduced infiltration of CD4 T lymphocytes and microglial activation was also reported [255]. Similarly, anandamide-reduced VCAM-1 expression in brain endothelial cell cultures through a CB1-dependent mechanism and possibly A2A receptors [256,257].

The complex action of cannabinoids in MS is also demonstrated in a study focused on the effect of THC+CBD combination in murine EAE. The improvement of clinical manifestations in this model was based on the reduction of neuroinflammatory processes mediated by CB1 and CB2 activation. Authors found reduced levels of Th1 and Th17 cells, decreased CD4+ T infiltrative cells into the brain, decreased pro-inflammatory molecules (IL-17, INF-γ, TNF-α, IL-1β, IL-6, and TBX21), and increased FoxP3, STAT5b, interleukin 4 (IL-4), interleukin 10 (IL-10), and TGF-β, and apoptosis. The effects were also mirrored by changes in miRNA profile in brain-infiltrating cells [258].

Growing evidence indicates that epigenetic regulation is involved in CBD immune modulation. In MOG-sensitized lymphocytes, different histone methylation levels in binding sites of certain transcription factors suggest that these may play important roles in CBD-mediated immune modulation. Abnormal expression patterns of various miRNAs with pro- and anti-inflammatory properties was counteracted by CBD. In transcriptome expression analysis, CBD suppressed 876 MOG-induced transcripts and induced 396 MOG-suppressed transcripts. These changes are known to be involved in cell cycle and immune response and are in line with previous results showing that CBD inhibits T cell proliferation [259].

Recent data from experimental studies focusing on innate immunity might provide new insights into MS physiopathology and may reveal new evidence of a potential causal relationship between MS and viral infection [260]. O’Brien and collaborators postulated that this relationship may represent the pillar of the progression of MS [261]. Furthermore, experimental studies have shown that signaling pathways involving the family of the toll-like receptors (TLR) may influence the progression of MS in animal models [262]. For example, the expression of the TLR3 and TLR 4 receptors is increased in active lesions [263]. A study in MS patients focused on the effects of cannabidiol and ∆9-THC on TLR3 and TLR4 from peripheral blood mononuclear cells revealed anti-inflammatory responses since reduced expression of IFN-β was documented in both groups. Additionally, a pronounced favorable effect was reported if cells were pretreated with a 1:1 association of THC:CBD compared to the effect of each of them alone [264].

Microglia fulfill the role of resident mononuclear phagocytes in the brain parenchyma. They are essential players in local homeostasis and defense. Microglia is maintained in a stable surveillant phenotype by various signals from healthy neurons and astrocytes [265]. Once stimulated, microglia switch to either the M1 proinflammatory state producing pro-inflammatory cytokines such as IL-1β, IL-12, IL-18, IL-6, TNF-α, or to an alternative M2 state which is associated with regeneration and repairing (subtype M2a), immunoregulation (M2b) or an acquired-deactivating phenotype (M2c) [266]. M2 state microglia release anti-inflammatory factors such as IL-10 and IL-4 and express higher levels of receptors associated with phagocytosis [265]. Activated M1 microglia produce endocannabinoids [267] which promote the M2 phenotypes mainly through CB2 receptors [266]. In human brain samples from MS patients, CB2 receptors were present in T-lymphocytes, astrocytes, and perivascular and reactive microglia while CB1 receptors were found mostly on cortical neurons, oligodendrocytes, oligodendrocyte precursor cells, macrophages, and infiltrated T-lymphocytes. Interestingly, CB2-positive microglial cells were found into active plaques while in the case of chronic plaques, they were identified at the periphery [268].

In vitro studies have shown that cannabinoids activate the phosphoinositide 3 kinase/protein kinase B/mechanistic target of rapamycine (PI3K/Akt/mTOR) pathway which is known to play a central role in the regulation of inflammation, cell survival and differentiation. This is mainly achieved through CB1 receptors leading to a protective effect on astrocytes [269] and oligodendrocyte progenitor cells [270]. Interestingly, in EAE mice study, cannabidiol was able to restore PI3K/Akt/mTOR function. Moreover, a reduction of pro-inflammatory cytokines such as IFN-γ and IL-17, an increased level of BNDF together with up-regulation of PPAR-γ and inhibition of c-Jun N-terminal kinase (JNK) and p38 mitogen-activated protein kinase (MAP) kinases were reported in the same study [271].

The dysfunction of the oligodendrocyte plays a major role in the physiopathology of MS and pharmacological interventions that specifically target their protection or regeneration may provide a promising approach towards their remyelination. It is clear now that oligodendrogenesis is not restricted only to developmental periods. In contrast, recent evidence showed that this process is a permanent part of brain activity [272,273]. Oligodendrocyte progenitor cells (OPC) differentiate into oligodendrocytes under the influence of a plethora of intrinsic and extrinsic factors [274]. Among them, the cannabinoid system may play an essential role in the modulation of this process. Both CB1 and CB2 receptors are found on oligodendrocytes and their precursors and cannabinoids have been shown to exert a protective effect that involves the activation of the PI3K/Akt signaling pathway [270]. Additionally, other endocannabinoid-dependent protective mechanisms are stimulated during inflammation such as the reduction of endoplasmic reticulum stress pathways-related apoptosis and the inhibition of LPS/IFN-γ induced phosphorylation of eiF2α and protein kinase R (PKR) [275]. Intriguingly, a dose-dependent relationship was documented since different doses of a cannabinoid drug may induce opposite effects. For example, exposure of cuprizone fed mice to CB1 agonist WIN-55,212-2 at a dose of 1 mg/kg aggravated demyelination and prevented OPC proliferation while a lower dose of 0.5 mg/kg had a protective and anti-inflammatory effect [276].

Unfortunately, the promising results from in vitro and in vivo studies are not yet fully paralleled by the results from observational studies or clinical trials and controversies still exist. For example, in a sub-study of CAMS (Cannabinoids in MS), no evidence for a benefic cannabinoid influence on serum levels of IFN-γ, IL-10, IL-12, or C-reactive protein was found. Moreover, mitogenic stimulation experiments also failed to demonstrate any significant reduction of CD3+ and IFN-γ production [277]. In contrast, a recent prospective case control study showed that the levels of pro-inflammatory cytokines such as IL-1, IL-2, IL-6, IL-17, IL-22, TNF-α, and IFN-γ were significantly increased in the MS group when compared to the MS/cannabis and control groups. Moreover, the same study showed that in the MS population, anti-inflammatory cytokines such as IL-4, IL-10, and interferon-β1 (IFN-β1) had significantly lower values [278].

Although MS is still challenging and is not completely understood in terms of causality and pathogeny, the ECS may represent a promising approach not only for the treatment, but also for the alleviation of MS-associated symptoms and/or for increasing the efficacy of existing drugs. Recent and growing evidence has shown that CS may have the potential to modulate virtually all the major processes in MS involving genetic expression, inflammatory reactions, cell survival and interaction. Moreover, the beneficial effects of cannabinoids in neurodegenerative diseases such as multiple sclerosis may result from their antioxidant properties, as well as their regulatory functions in the inflammatory responses mediated by ECS [279]. Though promising, further studies involving the ECS in MS are necessary to overcome the potential adverse effects, lack of specificity, and/or methodological issues.

## 4. The Endocannabinoid System and Aging-Related Musculoskeletal Changes

Aging is a physiological process associated with changes in bone density, decreased muscle mass and function, and joint degradation [280,281,282]. Whereas various risk factors and pathological mechanisms have been identified and described with respect to aging-related musculoskeletal changes, the potential role of endocannabinoid signaling is yet to be fully understood. Nevertheless, recent studies highlight the active involvement of ECS in bone metabolism, sarcopenia, and degenerative joint disease [283,284,285].

### 4.1. The Endocannabinoid System and Bone Changes

Older adults experience a marked decrease in bone density mainly due to a disruption in bone metabolism. In this respect, the imbalance between bone formation and resorption favoring the latter in aging individuals has been linked to endocrine factors, comorbidities, inflammaging, medications, low physical activity or bedrest, sarcopenia, increased bone marrow adipogenesis, and poor diet [286,287,288,289]. While osteopenia and osteoporosis are more frequently found in postmenopausal women, a notable aging-related decrease in bone density has also been described in men and some authors suggest that this is a largely underestimated issue in the older male population [290].

Studies have indicated that bone metabolism is significantly impacted by the activity of the ECS. The connection between ECS activity and bone mass is supported by recent findings describing the presence in bone tissue of ECS-related molecules such as the receptors CB1 and CB2, together with AEA, 2-AG, as well as the enzymatic equipment involved in endogenous cannabinoid metabolism. Moreover, the cannabinoid receptors CB1 and CB2 have been described as potential future therapeutic targets for osteoporosis [289,291].

**Figure 4:**
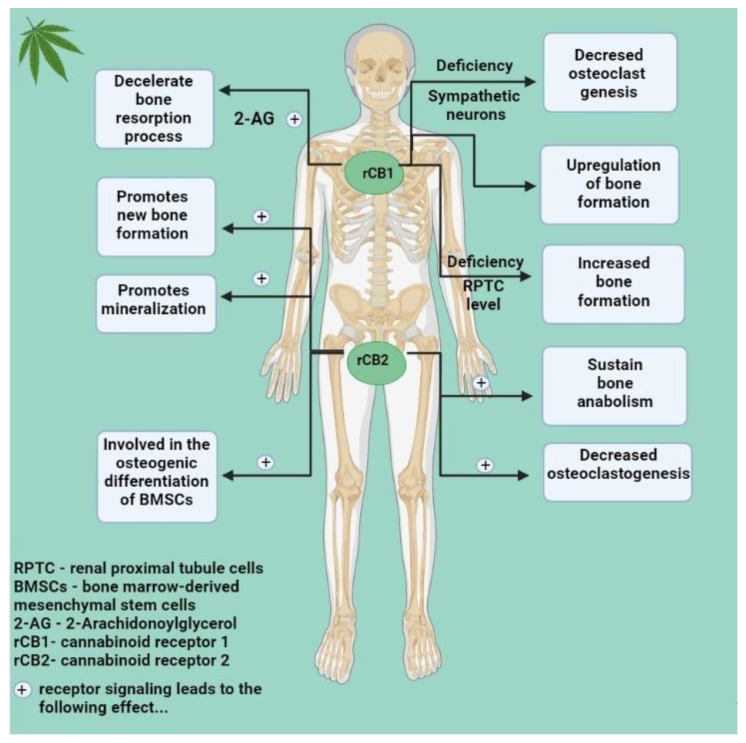
Schematic representation of the relationship between endocannabinoid system and bone changes.

The release of norepinephrine by sympathetic fibers leads to an enhancement in bone resorption and a decrease in bone formation. Through its effect on the sympathetic nervous system which includes the interruption of noradrenaline release, rCB1 stimulation by 2-AG has been shown to decelerate the process of bone resorption in mice [291]. The deterring effect of skeletal rCB1 on norepinephrine release followed by temporary stimulation of bone formation has been previously demonstrated in acute conditions. However, Deis et al. aimed to examine the potential feedback loop between sympathetic nerve fiber activity and bone-forming osteoblasts in chronic conditions. The evaluation included bone mass changes at the level of the distal femoral metaphysis and vertebrae of young rCB1 deficient (12 weeks old) and older male mice (35 weeks old) compared to age-matched controls. Although in young rCB1 null mice, there was no difference in bone mass compared to wild-type controls, in the aging CB1r-deficient animals, the authors noted an unexpected increase in bone mass. rCB1 deficiency at the level of sympathetic neurons was linked to an upregulation of bone formation and a decreased osteoclast genesis, indicating that rCB1 deletion interfere with the feedback circuit between ECS and sympathetic nerve activity with regard to bone formation [292]. The relationship between endocannabinoid system and bone changes is summarized in Figure 4.

The activity of renal proximal tubule cells (RPTC) impacts bone metabolism. These cells express rCB1 (RPTC-CB1r), the latter influencing the regulation of bone mass. Baraghithy et al. found that rRPTC-CB1 deficient mice led to an increased bone mass phenotype (higher trabecular bone volume ratio in the distal femoral metaphysis—BV/TV, with an augmented trabecular thickness and number) and greater bone mineral density compared to the control group. In rRPTC-CB1-/- mice, the authors noted an enhancement of osteoblast activity linked to an increased bone formation. Interestingly, a higher number of osteoclasts per trabecular perimeter was also identified in null mice, yet without a corresponding augmentation of osteoclast activity [293].

According to a number of animal studies, rCB2 signaling has the ability to sustain bone anabolism. rCB2-deficient mice may exhibit age-related trabecular and cortical bone changes similar to postmenopausal osteoporosis. Additionally, decreased bone strength and lower bone mineral density were linked to polymorphisms in the coding region of rCB2 in human subjects, suggesting that ECS-related genetic factors may contribute to the risk of osteopenia or osteoporosis [291].

rCB2 signaling was shown to either promote new bone formation and mineralization or decrease osteoclastogenesis [294]. However, in cultured murine macrophages, Li and Sun found that the rCB2-selective agonist AM1241 boosted receptor activator of nuclear factor kappa-B ligand (RANKL) dependent osteoclast differentiation, whereas the selective antagonist AM630 hampered this process [295].

Bone marrow-derived mesenchymal stem cells (BMSCs) are multipotent cells which have the ability to differentiate into osteoblasts. Moreover, these cells express rCB2 [296]. rCB2 is believed to be involved in the osteogenic differentiation of BMSCs. Cultured BMSCs isolated from the bone marrow samples of healthy donors and patients with osteoporosis revealed a decreased expression of CB2r in osteoporotic subjects compared to the control group. The study conducted by Wang et al. focused on analyzing the potential role of rCB2 signaling in the restoration of osteogenic differentiation of BMSCs sampled from human subjects with osteoporosis. The overexpression of rCB2 in human osteoporotic BMSCs boosted alkaline phosphatase (ALP) activity, favored osteogenic gene expression and increased mineralized extracellular matrix deposition [297].

Sophocleous et al. examined the effects of double rCB deficiency (rCB1 and rCB2) on bone development (from birth to aging mice) and studied its impact on ovariectomy-induced bone loss in female mice. rCB1/CB2 deficiency in mice led to an osteoclast defect and a subsequent protective effect on ovariectomy-induced and age-related bone loss in experimental animals. Moreover, the positive effect of reduced osteoclast number surpassed the negative impact of the observed reduction in bone formation leading thus to the preservation of bone mass. These findings were not paralleled by those obtained from the distinct inactivation of rCB1 and rCB2, suggesting that the two receptors have coinciding (but not redundant) effects on bone mass in aging experimental animals and ovariectomized female mice [298].

AEA and 2-AG may be produced at the level of trabecular bone, as shown in animal studies. Additionally, AEA and 2-AG were found to be produced by human osteoclasts. Moreover, experimental studies have shown that 2-AG treatment of rodent BMSCs resulted in an increase of ALP which is known as a marker of osteoblast differentiation. However, other research studies did not report an increase of ALP in murine osteoblast cell lines [294]. Smith et al. examined the effects of endocannabinoids AEA and 2-AG on cultured human osteoblast activity. At four days, AEA and 2-AG prevent osteoblast differentiation with a concentration-related augmentation of ALP levels. Overall, AEA was linked to early osteoblast differentiation, while 2-AG was associated with an early increase and a late decrease in the levels of osteoblast differentiation biomarkers [299].

Two endocannabinoid-like molecules, palmitoylethanolamide (PEA) and oleoylethanolamide (OEA), were studied with respect to a variety of processes including endocannabinoid tone during osteoblastic differentiation. Kostrzewa et al. analyzed this relationship in a murine osteoblast cell line MC3T3-E1 and found that during osteoblastic differentiation, the levels of OEA increased while AEA and 2-AG declined in maturing and mineralized cells. In the femurs of male mice, there was a notable age-related reduction in PEA, OEA and 2-AG expression [294].

Monoacylglycerol lipase, a lipolytic enzyme which is known to degrade the endogenous cannabinoid 2-AG, has been described as a potential therapeutic target for osteoporosis due to its presumed impact on osteoclast differentiation. Interestingly, during osteoclast differentiation the monoacylglycerol lipase protein expression may be augmented. Moreover, Liu et al. found that monoacylglycerol lipase deletion in bone marrow-derived macrophages was linked with the inhibition of both bone resorption and osteoclast genesis [300].

### 4.2. The Endocannabinoid System and Osteoarthritis

Osteoarthritis (OA) is the most prevalent joint disease in aging individuals and is mainly characterized by a progressive degeneration of articular structures. Whereas other changes such as bone remodeling and synovial inflammation have also been described in osteoarthritic joints, cartilage degradation plays a central role in the pathogenic process [301].

A large number of studies focusing on analgesia have described the pain as the most prominent symptom of the disease and also the ability of the ECS to modulate pain in OA [302]. Mlost et al. found that β-caryophyllene (a natural low-efficacy agonist of CB2r that is present in human diet) had both antinociceptive and chondroprotective effects in animals with experimentally induced osteoarthritis. Additionally, chronic administration of β-caryophyllene reduced cartilage degradation without inducing tolerance to its analgesic properties [303].

In wild-type mice, the selective rCB2 agonist HU308 developed beneficial effects on knee osteoarthritis secondary to a surgically-induced destabilization of the medial meniscus [304]. The examination of cultured chondrocytes from rCB2-deficient experimental animals revealed that these cells produced lower amounts of proteoglycans compared to chondrocytes obtained from wild-type mice, indicating a role for rCB2 signaling in the development and progression of OA in mice [305].

Bryk et al. conducted a study on rodents with monoiodoacetate-induced knee OA and investigated the gene expression of ECS constituents at the level of the spine and knees of the experimental animals during the early and late stages of osteoarthritis development. The authors found an enhancement of AEA synthesis and degradation enzymes in the early phases of OA. There was no upregulation of rCB1 and rCB2 gene expression in the rats’ cartilage. Also, the transcript levels of AEA synthesis and degradation enzymes did not vary significantly in the animals’ cartilage. However, in the synovial membrane samples, Cnr2 gene expression started to increase from day 2 after monoiodoacetate injection and was significantly upregulated after 2 weeks up to the end of the study (14 to 28 days after injection). N-arachidonoyl phosphatidylethanolamine phospholipase D (Nape-plD) gene expression was augmented from the second day of the experiment. Both the cartilage and synovial membrane samples exhibited modifications in the transcript levels of the AEA alternative synthesis and degradation pathways. Nonetheless, more changes were seen in the synovial membrane [306].

Gaisberger et al. recruited elderly patients with knee OA who underwent spa treatment with or without low-dose radon therapy for 2 weeks. Plasma AEA levels were measured at baseline and after 2 weeks. AEA values were significantly decreased post-treatment and were paralleled by pain reduction in both study groups. No relationship between AEA and the tested potential serum and urinary markers of cartilage degradation was described [307].

### 4.3. The Endocannabinoid System and Skeletal Muscle Changes

Aging-related sarcopenia includes a marked decrease in both muscle mass and function compared to young adults and is frequently accompanied by different degrees of disability [308]. Whereas particular ECS constituents have been associated with age-dependent muscle changes, the impact of endocannabinoids on muscle mass and function over time requires further investigations.

Le Bacquer et al. aimed to describe the relationship between endocannabinoids and muscle-related parameters in young adult versus older male Wistar rats. The authors evaluated the levels of endocannabinoids AEA and 2-AG, and endocannabinoid-related N-acylethanolamines PEA and OEA in the animals’ plasma, skeletal muscles (oxidative and glycolytic), and adipose tissue. While the subjects’ weight did not differ, the body composition of the older rats revealed a higher fat mass and a lower lean mass. Sarcopenic animals demonstrated impaired motor activity and significantly lower plasma levels of PEA and AEA. In rats with sarcopenia, the authors found a marked augmentation of 2-AG levels in the soleus muscle (oxidative) and a low OEA expression in the extensor digitorum longus muscle (glycolytic). Elevated levels of AEA, PEA, and OEA were described in the sarcopenic animals’ subcutaneous fat. Moreover, the abovementioned changes were accompanied by altered ECS-linked gene expression in both the skeletal muscles and the adipose tissue of older rats compared to young adults [284].

Dalle and Koppo examined the expression of cannabinoid receptors in young men (20–27 years of age) versus old non-sarcopenic males (65–84 years of age) and found that rCB1 expression in the vastus lateralis muscle was bigger in aging individuals while rCB2 expression did not significantly differ. In healthy older adults (physically active, non-sarcopenic participants over 65 years of age, males and females), a 12-week resistance exercise program increased both rCB1 and rCB2 expression at the level of the vastus lateralis muscle but without statistical significance. Intriguingly, in older participants of both sexes, the authors identified statistically significant correlations between the difference in rCB2 expression post- versus pre-resistance training and the levels of muscle maintenance markers (FOXO3a and the myogenic markers MyoD and Pax7) [309].

In the past decade, a growing body of evidence has paved the way for ascertaining a relationship between ECS constituents and endocannabinoid-like molecules in various aging-associated musculoskeletal changes [301,303,306,309]. Most scientific evidence pertaining to this relationship derives from in vitro and in vivo studies and the potential applicability of these findings to the management of osteoporosis, osteoarthritis or sarcopenia in human subjects require further studies. Nevertheless, recent and encouraging studies provide valuable insight into the involvement of ECS in the musculo-skeletal changes associated with aging.

## 5. Cannabinoid Implications in Age-Related Oncological Diseases

Since the median age of cancer diagnosis is around 66 years, this disorder is now considered the disease of aging [310]. The advances in oncological disease prevention strategies together with the improvements in healthcare systems will shift towards a greater incidence and prevalence the neoplasic conditions in the elderly. According to the World Health Organization (WHO), in the majority of developed countries in which life expectancy is now exceeding 80 years, the prevalence of cancer is projected to increase by 45% until 2030 [311,312]. Thus, various strategies of the healthcare systems focusing on the improvement of the prevention and treatment effectiveness to cure or ameliorate the quality of life of oncological patients are mandatory.

Among many molecular pathological pathways involved in the development of cancer disease, recent evidence has shown that the cellular senescence may play an important role. As mentioned before, senescent cells have the ability to secrete pro-inflammatory cytokines and growth factors (the SASP phenotype) which are known to induce and maintain a chronic inflammatory state. Together with their ability to provide a supportive environment for the development and progression of cancer cells, senescent cell targeting may be a promising approach to improve the management of cancer conditions [313,314].

Recent experimental and clinical studies focusing on identifying novel and promising therapeutic approaches addressed to both oncological conditions and cancer-related disorders have described a potential benefit of the endocannabinoid system. It is already known that cannabinoids are indicated to ameliorate cancer related pain, chemotherapy-induced nausea and vomiting, cachexia, or anorexia [315]. However, recent studies suggested that dysregulation of the ECS may also promote cancer development by fostering physiological conditions which allow cancer cells to proliferate and migrate. Clearly, targeting this mechanism with various natural or synthetic compounds may have the theoretical potential of an improved control of cancer progression. Moreover, in the past years many authors proposed the hypothesis that the endocannabinoid system may be used not only as a prognosis marker, but also as a marker of carcinogenesis.

### 5.1. The Relationship between the Endocannabinoid System, Carcinogenesis, and Tumor Progression

Experimental studies have shown that both rCB1 and rCB2 agonists induce anti-proliferative and pro-apoptotic effects most likely through the downregulation of gene transcription and the increase of intracellular ceramide [316]. The mechanisms responsible for cannabinoid-induced autophagy are still under debate, some authors proposing the inhibition of protein kinase B/the target of rapamycin kinase complex 1 (Akt/mTORC1) axis [317], while others suggest that upregulation of cyclooxygenase 2 (Cox-2) and prostaglandin E-2 (PGE2) are more probably involved [318].

**Figure 5:**
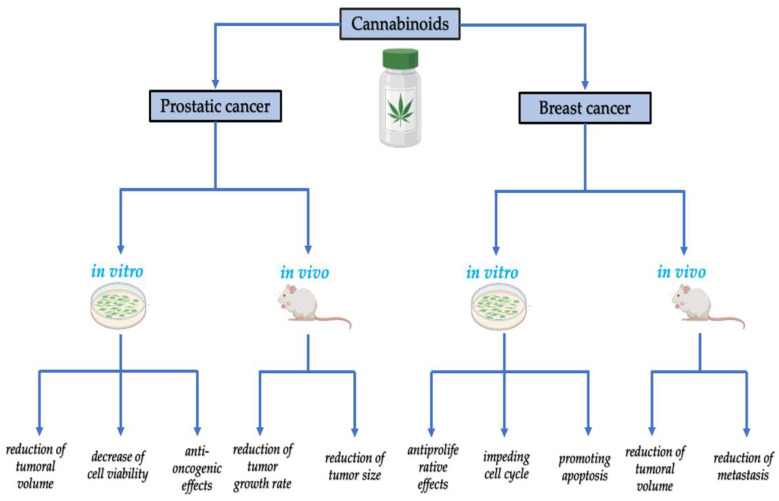
Schematic representation of the effects of cannabinoids in prostatic and breast cancer.

Several studies have focused on evaluating the antineoplastic effects of cannabinoids in geriatric patients. For example, in vitro studies using prostatic cancer cells sampled from patients, cannabinoids administration in various concentrations caused a decrease of the tumoral volume by the induction of apoptosis, decrease in cell viability or interfering with different cell signaling pathways which counteract oncogenesis [319,320,321,322,323,324]. Interestingly, further in vivo studies have confirmed the antitumoral effect of cannabinoids since encouraging results such as reduction in the rate of growth and size of the tumors were reported [321,322]. The same antiproliferative effects were also reported in breast cancer together with the reduction of cell viability, alteration of signaling pathways and impeding cell cycle and promoting apoptosis [325,326,327,328,329,330]. Reduction of both tumoral volume and metastases have been also reported in in vivo studies [330]. Moreover, in a recent paper aiming to evaluate the cancer risk in cannabis users, Clark et al. reported that several types of head and neck cancer occur less frequently in this population suggesting that the ECS may represent a promising approach for the management of cancer [331]. The main effects of cannabinoids in prostatic and breast cancer are summarized in Figure 5. 

The ECS has also been linked to essential events in the cascade of cancer progression such as invasion, metastasis and angiogenesis [318]. For example, in various types of solid tumors such as glioblastoma, prostate cancer, endometrial sarcoma, and colon cancer, increased concentrations of AEA and 2-AG have been reported when compared to non-cancerous tissues suggesting thus a possible endocannabinoid control mechanism of cancer growth [332]. Furthermore, in animal models of breast cancer, peritumoral administration of CBD induced a decrease in the recruitment of tumor-associated M2 pro-tumorigenic macrophages type [333]. This suggests that cannabinoids may have the ability to modulate tumoral microenvironment by lowering the CC chemokine ligand 3 (CCL3) levels reducing, and thus the macrophage chemotactic activity and metastasis induction [334,335,336].

Recent evidence has shown that the variance of CB1 and CB2 receptors expression may impact the prognosis of the disease, as summarized in Table 4. For example, in tissular hepatocarcinoma samples, overexpression of both CB1 and CB2 receptors was correlated with a decreased likelihood of portal vein invasion and a subsequent improvement in disease-free survival rate [337]. An inverse correlation between rCB2 expression and tumor aggressiveness was also reported for gliomas [338]. Moreover, in non-small cell lung cancer patients, a statistically significant positive correlation was described between an increased CB1 and CB2 receptor expression and survival rate [339]. The association between rCB1 and disease severity and outcome has been also documented in prostatic cancer. Specifically, a higher expression of the cannabinoid receptor in tumoral tissue was correlated with an increased Gleason score and the presence of metastases at diagnosis [340]. Furthermore, patients with a high CB1-immunoreactivity (CB1-IR) score had shorter disease-specific survival rate [341]. In another study focusing on malignant and non-malignant thyroid cells, an increased rCB2 expression was correlated with a high recurrence rate, lymphatic and vascular invasion, and lymph node metastasis [342]. Similarly, a study on human pancreatic ductal carcinoma samples suggested that there is an inverse correlation between rCB1 levels and survival rate [343]. The impact of cannabinoids receptor expression on cancer development and their implication on the disease prognosis is summarized in Table 4.

### 5.2. Cannabinoid Usage for the Management of Pain-Associated Cancer

Various aging-associated comorbidities such as dementia, arthritis, and osteoporosis may challenge the proper management of pain in the elderly oncological population and often such patients may receive an inadequate antalgic treatment [348,349]. Recent evidence has shown that ECS may be considered a promising approach since encouraging results from clinical and experimental studies were reported. Several molecular mechanisms for the analgesic effect of CBD have been proposed and studied in animal models. For example, in neuropathic and taxol-induced pain animal models, CBD showed favorable analgesic effects [350]. Although some experimental studies stated exciting results, clinical trials still report conflicting effects. In a randomized, double-blind study focusing on the effects of topical CBD on neuropathic pain, the authors reported more beneficial effects when compared to placebo [351]. Moreover, two randomized, placebo-controlled trials have shown that in patients with an inadequate analgesia control despite the use of opioids, Nabiximols significantly reduces cancer-related pain when compared to placebo [352,353]. On the other hand, a randomized study investigating the potential antalgic effect of Nabiximols for chemotherapy-induced neuropathic pain reported no significant benefits when compared to placebo [354]. Clearly, further experimental and clinical studies are required to find novel ECS-related molecules which may interact with various pathways involved in cancer-associated pain to improve the quality of life of oncological patients.

### 5.3. Cannabinoid Usage to Ameliorate Chemotherapy-Induced Nausea and Vomiting

In the cancer population, observational studies have reported an increased frailty index which is a predictor of mortality and morbidity related to chemotoxicity [355,356]. Additionally, in such patients, an increased incidence of chemotherapy-induced organ toxicity was also described which frequently requires the lowering of the chemotherapeutic doses to ameliorate this undesirable effect.

Chemotherapy-induced emesis significantly influences the quality of life leading usually to anorexia and metabolic disorders which may negatively impact the administration of anticancer drugs. Currently, two cannabinoid drugs are approved for the usage in clinical practice, while dronabinol is indicated as a first line drug for the treatment of chemotherapy-induced emesis, nabilone is recommended in patients who have failed to respond to conventional antiemetic treatments [357]. Interestingly, cannabinoid administration in such patients reduced the emetic reflex by inhibiting the release of excitatory neurotransmitters, mainly through rCB1 dependent mechanisms [358]. Furthermore, in a large multicenter randomized placebo-controlled trial, oral THC:CBD cannabis extract successfully ameliorated symptoms induced-chemotherapy such as nausea and vomiting when compared to standard antiemetic therapy, although more side effects were reported in THC:CBD group [359]. Moreover, in a phase II clinical trial including 16 patients, 4.8 sprays of Nabiximols were more effective than placebo in further reducing chemotherapy-induced nausea and vomiting in patients on standard antiemetics [360].

### 5.4. Cannabinoid Usage to Ameliorate Cancer—Associated Cachexia and Anorexia

As before mentioned, a common feature of aging is sarcopenia, which represents the physiological loss of both muscle mass and function [361]. In cancer patients, the muscle loss is usually accelerated leading to cachexia which is associated with a functional impairment, reduced physical performance and a decreased survival rate. Particularly, if cachexia occurs in patients that are already sarcopenic, an increase of the risk of malnutrition and deleterious side effects is commonly reported [362]. The ECS may have an important role to ameliorate cancer associated cachexia since cannabinoids are known to modulate eating behavior. Early reports showed a benefit of Dronabinol on the appetite and weight stability of HIV/AIDS patients [363]. However, the data supporting cannabinoids for cancer cachexia are limited. In a clinical study, Jatoi et al. showed that Megestrol acetate provided superior anorexia palliation among advanced cancer patients when compared to dronabinol alone (75% versus 49% for appetite and 11% versus 10% for baseline weight gain). Unfortunately, the administration of both drugs did not show additional benefits [364]. Although cannabinoids must be used with caution in the geriatric population due to the risk of delirium, growing evidence supports the hypothesis that these drugs may be considered for use in daily clinical practice to ameliorate cachexia-associated cancer in patients with life expectancy of days to months.

### 5.5. Cannabinoid Usage to Ameliorate Cancer—Associated Anxiety and Depression

Observational studies have reported poorer treatment outcomes, decreased compliance, prolonged hospitalization, and suicide in cancer patients in which the rate of both anxiety and depression is up to 23% and 26%, respectively [365,366,367,368,369]. It is clear now that the treatment against anxiety and depression is mandatory in such patients to improve not only the quality of life but also the overall prognosis. Although the role of CBD as an anxiolytic agent has been intensively studied in a variety of diseases such as generalized anxiety disorder, social phobia, and schizophrenia, a limited number of studies assessed the effects of cannabinoids on cancer associated anxiety or depression. Good et al. investigated the effects of cannabinoids in palliative care and reported a significant reduction of anxiety and depression. Moreover, the same study reported a decrease in the median depression, anxiety, and stress scale (DASS 21) scores, with the biggest reduction of depression and stress [370].

The modulation of the ECS which may counteract the processes involved in cancer development such as the promotion of cell autophagy and apoptosis, antiproliferative effects, or reduction of metastasis outgrowth is an attractive and promising pharmacological intervention. Although encouraging results are reported from both experimental and clinical studies, further investigations are required to confirm all these benefits of cannabinoids on ameliorating or even cure cancer associated various symptoms such as nausea, vomiting, anxiety, or depression.

## 6. Cardiovascular Aging and Cannabinoid System 

Cardiovascular diseases (CVDs) represent the leading cause of mortality globally with recent epidemiological studies showing an increasing trend of prevalence and incidence. Since in the next decade it is estimated that one-fifth of the world’s population will be over 65 years and giving that aging has the greatest impact on cardiovascular homeostasis, it is a certainty that the global burden of cardiovascular diseases will increase [371,372]. Thus, emerging non-pharmacological and pharmacological therapies are mandatory to reduce the burden of CVDs.

Growing evidence suggests that ECS may play an important role in the modulation of cardiovascular parameters such as blood pressure, vasomotor tone, cardiac contractility, vascular inflammation, and angiogenesis [373]. Interestingly, in various aging associated cardiovascular diseases such as obesity, diabetes, and hypertension, dysregulation of the ECS signaling was shown to play pivotal roles. Moreover, a decreased plasma levels of 2-AG and AEA is associated with the alteration of cannabinoid receptor expression in the vasculature, suggesting that the ECS may represent a promising complementary therapeutic approach for the treatment of aging associated cardiovascular diseases [374,375,376,377,378]. We will further focus on describing the relationship between the ECS modulation and the physiopathology of the two most prevalent risk factor for CVDs associated with aging: arterial hypertension and atherosclerosis.

### 6.1. The Endocannabinoid System and Aging—Associated Hypertension

Among the risk factors for CVDs, arterial hypertension has the highest prevalence and is the major cause for heart failure, atrial fibrillation, coronary heart disease, stroke, chronic kidney disease, and dementia. Since aging is strongly correlated with an increase in arterial blood pressure values, it is expected that the prevention of age-related increase in blood pressure would decrease the vascular consequences commonly attributed to aging [379].

Although a plethora of antihypertensive drugs are currently available, a proper management of arterial hypertension is still challenging to be achieved. For example, observational studies reported a prevalence of resistant hypertension between 10 and 25% in the general population and up to 12% in elderly patients [380]. Interestingly, the ECS may play a major role in the physiopathology of arterial hypertension since significant antihypertensive effects of cannabinoids, their endogenous and synthetic analogs were reported. Among the mechanisms of action, NO-related pathways, CB1, and TRPV1 receptors were postulated to be involved [381,382]. Moreover, one of the endogenous cannabinoids’ metabolism is arachidonic acid which is further converted in vasoactive products and modulate the regulation of vascular tone, local blood flow, and blood pressure [383].

Experimental studies have suggested that AEA may play a pivotal role in the physiopathology of arterial hypertension since AEA treatment has been shown to induce various antihypertensive effects in in vivo [382,384,385]. For example, AEA administration led to a transient decrease of blood pressure and heart rate due to the activation of the vanilloid TRPV1 receptors from sensory vagal neurons in the heart. Moreover, through rCB1 found in heart and blood vessels which are involved in counteracting the production of noradrenaline, AEA is able to reduce the cardiac contractility and peripheral resistance [386]. Intriguingly, AEA is also able to modulate vascular tone through the release of nitric oxide (NO) and endothelium-derived hyperpolarizing factor (EDHF) from the vascular endothelium [387]. Furthermore, ECS is also postulated to be involved in the long-term control of blood pressure since antihypertensive effects were linked to AEA and COX-2 metabolites from renal medulla. For example, AEA infusion into renal medulla promotes diuresis and natriuresis due to NO generation and inhibition of Na+/H+ and Na+/K+/2Cl cotransporters through the rCB1 [388,389,390]. These findings suggest that an increased AEA concentration at various levels which may be achieved by the inhibition of the enzymatic breakdown or cellular uptake may represent a promising therapeutic approach for the treatment of arterial hypertension. Thus, the development of active drugs targeting AEA pathways may complement the traditional lowering blood pressure therapy.

### 6.2. The ECS and Aging—Associated Atherosclerosis

Atherosclerosis is a chronic arterial disease and represent the leading cause of vascular death globally due to cardiovascular and neurological complications such as ischemic heart and peripheral disease and stroke [391]. Recent evidence has shown that atherosclerosis is a systemic inflammatory disease in which the accumulation of lipids into arterial wall is mirrored by a pro-inflammatory state driven by chemokines and leukocytes leading to the formation of atherosclerotic plaque [392]. Atherosclerosis is also known as a disease of aging and the interplay between increasing age and atherosclerosis is still under research. It is clear now that aging is an independent risk factor for the development of atherosclerosis and the cellular senescence is the main factor which promote atherosclerosis [393].

**Figure 6:**
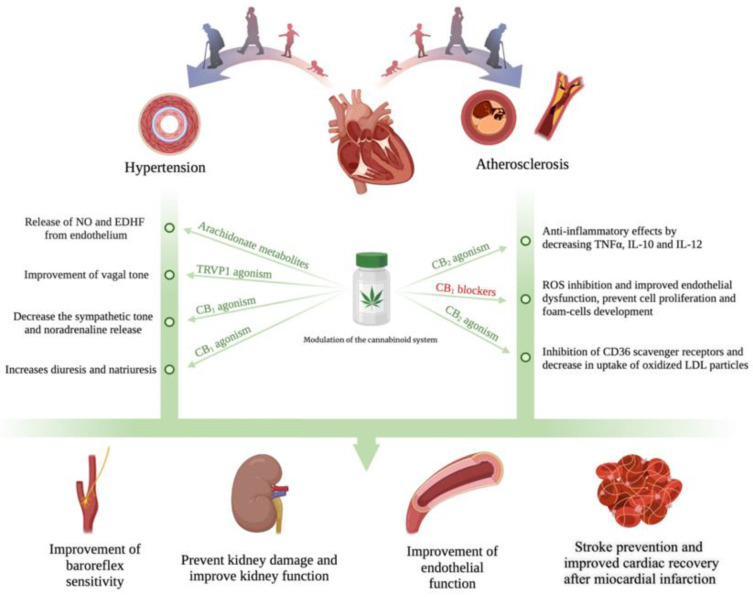
Schematic representation of the main effects of cannabinoids in hypertension and atherosclerosis (NO—Nitric Oxide; EDHF—endothelium-derived hyperpolarizing factor; TRVP1—transient receptor potential vanilloid 1; CB1—cannabinoids receptor 1; CB2—cannabinoids receptor 2; TNF-α—Tumor Necrosis Factor Alpha; IL-10—Interleukin 10; IL-12—Interleukin12; ROS—Reactive Oxygen Species; CD-36—Cluster of Differentiation 36; LDL—Low Density Lipoproteins).

Clinical and experimental evidence has reported high levels of both local and systemic endocannabinoid levels paralleling local atherogenic lesions or systemic atherosclerosis. For example, in patients with coronary artery disease, increased levels of systemic endocannabinoids were reported [394]. Montecucco et al. also found high levels of endocannabinoids and an increased expression of both rCB1 and rCB2 in human carotid plaque samples [125]. Moreover, in an animal model of atherosclerosis, Steffens et al. have reported lower dimensions of atherosclerotic plaques and reduced local inflammation in mice treated with THC. Interestingly, these favorable effects were mitigated by the administration of rCB2-selective antagonists, suggesting that the anti-atherosclerotic effects induced by THC are modulated through rCB2 pathways [395].These findings are in line with another experimental study on LDL receptor knockout mice (LDLr^−/−^) which demonstrated that rCB2-deletion increased macrophage infiltration in atherosclerotic lesions [396]. Furthermore, Chiurchiu et al. demonstrated that the treatment of human-derived foam cells with rCB2 agonist have anti-inflammatory effects since it was able to significantly lower TNF- α, IL-10, and IL-12 levels. They also reported a reduced expression of the CD36 scavenger receptor which is known to be involved in the uptake of oxidized LDL during foam cell production [397]. Although the CB2 receptor pathway clearly has favorable effects in the physiopathology of atherosclerosis, rCB1 seems to have opposite mechanisms of action since their activation led to ROS generation and induction of apoptosis in primary human coronary artery endothelial cells [398]. Interestingly, an increased expression of rCB1 was also reported in vulnerable atherosclerotic plaques in which a high level of activated immune cells is usually found, suggesting that CB1 pathway may be involved in the modulation of inflammatory processes during plaque development [399]. This hypothesis is supported by the experimental results showing that CB1 pathway inhibition improved endothelial dysfunction, prevented cell proliferation, and foam-cells development and induced arterial vasodilatation [399,400]. The main effects of cannabinoids in hypertension and atherosclerosis are summarized in Figure 6.

Taken together, these findings suggest that selective rCB2 activation and rCB1 inhibition may have promising anti-atherosclerotic effects. Additionally, these encouraging results pave the way for further experimental and clinical studies to develop novel therapeutic agents which may have additional benefits to those of classical therapy currently used for the treatment of atherosclerosis.

## 7. Study Limitations

Although we have tried to emphasize the beneficial effects of ECS on various pathological mechanisms involved in multiple aging-related diseases, a series of limitations of this comprehensive review must be also discussed. For example, the contribution of genetic factors, as well as various influences from living environment which definitely influence the physiology of aging were not sufficiently take into account in this review [401,402]. In addition, various drug formulation and dosage as well as multiple adverse psychiatric reactions may also be considered as limitations of this study [403,404]. Therefore, from a pharmacokinetic point of view, cannabinoids are difficult to be managed in both animal and human studies since their absorption may vary. Furthermore, during chronic administration, cannabinoids may lead to drug tolerance which usually requires the increase of daily dosage with subsequent rise of psychiatric secondary effects [405]. These limitations hamper the translation of the results from experimental studies to daily clinical practice.

## 8. Conclusions and Perspectives

The cannabinoid system has the potential to ameliorate different underlying mechanism involved in the progression of aging-related diseases. Additionally, ECS may represent a promising approach not only for the treatment, but also for the alleviation of age-related disorder-associated symptoms and/or for increasing the efficacy of existing drugs. Moreover, our findings show that cannabinoids may be able to modulate various mechanisms rather than targeting a single dysregulated pathway in age-related diseases. Natural as well as synthetic cannabinoids ameliorate the balance between neurodegeneration and neuroinflammation in neurodegenerative diseases. In addition, they may play an important role in modulating the complex physio-pathology of MS and may be used as immune modulators, neuroprotectors, or remyelination promoters. The modulation of pro-inflammatory cytokines through the endogenous cannabinoid system may have beneficial effects on MS, AD, PD, aging-related musculoskeletal changes, and CVDs. On the other hand, it is clearly now that targeting the ECS with various natural or synthetic compounds may have the theoretical potential of an improved control of cancer progression.

Although a plethora of scientific advances on the functional relevance of cannabinoids in age-related diseases was recently achieved, a series of outstanding research questions still remain. We strongly believe that further experimental studies are mandatory to encourage the translational approach to clinical trials assessing the therapeutic potential of cannabinoids in various aging-related diseases.

## Figures and Tables

**Table 1:** The most important modulators of cannabinoid receptors, their mechanism of action, and the main findings in various experimental models.

Cannabinoids	Cannabinoid Origin	Mechanism of Action on Cannabinoids Receptors	Experimental Model	Main Findings	Ref.
**CP 55.940**	→ synthetic	→ rCB1 and CB2 agonist	→ acute pain models in mice; → neuropathic pain models induced by paclitaxel in CB1, CB2 knockout and wild-type mice.	→ CP 55.940 combined with a μ opioid agonist displayed synergism in two experimental pain test; → antiallodynic dose- dependent effects.	[14,15]
**Rimonabant** **(SR-141716A)**	→ synthetic	→ rCB1 selective → antagonist	→ obese Zucker (fa/fa) rats model; → non-obese Wistar rats.	→ reduction of hyperinsulinemia; → dose-dependent reduction of both food intake and body weight.	[16,17]
**AM251**	→ synthetic	→ neutral antagonist	→ acute foot-shock stress in mice; → neurodevelopmental animal model based on a social isolation procedure; → 129/SVE and C57BL/6 male mice.	→ antidepressant-like effect through interaction of opioid and cannabinoid pathways; → long-lasting effect on psychotic-like symptoms; → reduction in neuronal activity induced by isolation; → antidepressant-like and anorectic effect.	[18,19,20]
**WIN 55212-2**	→ synthetic	→ rCB1/CB2 → agonist with a slightly higher CB2R selectivity	→ neuropathic pain models; → autoimmune encephalomyelitis model; → behavioral changes induced by trauma exposure; → post-traumatic stress disorder models; → animal model of hypoxia-ischemia in fetal lambs.	→ analgesic effect; → reduction of the increased leukocyte rolling and firm adhesion in the brain; → restoring normal social behavior by modulating the stress response; → reduction of anxiety-like behavior; → reduction of the pro-inflammatory cytokines tumor necrosis factor (TNF)-α and interleukin (IL)-1β and IL-6 (interleukin 6).	[21,22,23,24,25]
**Δ9-THC (Δ9-tetrahydrocannabinol)**	→ natural	→ rCB1/ CB2 → partial agonist	→ male Sprague–Dawley rats exposed to chronic treatment with Δ9-THC.	→ neuroadaptive responses to cannabinoids via the increased expression of brain-derived neurotrophic factor.	[26,27]
**CBD** **(cannabidiol)**	→ natural	→ rCB1 antagonist/ inverse agonist; CB2 partial agonist	→ Alzheimer’s disease mouse model; → rat model of neuropathic pain; → epilepsy mice model; → Parkinson’s disease rat model.	→ reversed cognitive deficits in object recognition memory and social recognition memory; → modulates chronic neuropathic pain and depression-specific behavior; → reduces seizures and associated behavioral comorbidities; → neuroprotective and symptomatic effects.	[28,29,30,31]
**JWH-015**	→ synthetic	→ rCB2 selective → agonist	→ mice model of induced neuropathic pain; → rat model of induced arthritis; → acute and persistent inflammatory pain model.	→ antiallodynic effect; → anti-inflammatory effect; → antinociceptive effects.	[32,33,34]
**JWH-133**	→ synthetic	→ rCB2 selective → agonist	→ rat model of spinal cord ischemia reperfusion injury; → wild-type and knockout mice lacking CB2 in neurons, monocytes or constitutively, exploring spontaneous neuropathic pain.	→ attenuated neurological deficit and blood-spinal cord barrier disruption via toll-like receptors 4 (TLR4)/matrix metalloproteinase 9 (MMP9) signal pathway; → alleviates spontaneous pain and anxiety-associated behavior.	[35,36]

**Table 2:** The most representative clinical studies focusing on cannabinoids effect in various age related-diseases.

Cannabinoid	Specific Cannabinoid Studied	Disease	Number of Patients Enrolled	Inclusion Criteria	Main Findings	Ref.
**Synthetic** **Δ9-tetrahydrocannabinol**	Dronabinol	Multiple Sclerosis	240	→ McDonald criteria [61]; → age: 18–70 years old; → stable multiple sclerosis (MS) symptoms; → moderate to severe central neuropathic pain (CNP).	→ clinically significant reduction of mean pain intensities but without reaching a statistical significant difference between treatment and placebo groups; → dronabinol is safe over the long term; → positive influence on patients’ overall Quality of Life (QoF).	[62]
**Synthetic** **Δ9-tetrahydrocannabinol**	ECP002A (oral formulation of Δ9- tetrahydrocannabinol)	Multiple Sclerosis	24	→ age: ≥18 years old → progressive MS according to the revised McDonald criteria; → disease >1 year → clinically stable for at least 30 days before the inclusion; → moderate spasticity as defined by an Ashworth score of ≥2 (range, 0–4) and a Kurtzke Expanded Disability Status Scale score between 4.5 and 7.5 at baseline (range, 0–10).	→ ECP002A is well tolerated with a stable pharmacokinetic profile; → significant reduction of pain after ECP002A administration; → subjective MS related spasticity improved after 2 and 4 weeks of treatment.	[63]
**Synthetic** **Tetrahydrocannabinol:Cannabidiol spray**	Nabiximols	Multiple Sclerosis	106	→ age: ≥18 years old; → moderate to severe MS spasticity defined as a score of ≥4 on the MS spasticity 0–10 numerical rating scale; → spasticity symptoms for at least 12 months; → resistant MS spasticity.	→ THC:CBD spray significantly improved resistant MS spasticity when compared with first-line medication alone; → THC:CBD spray was significantly superior to placebo for spasms severity, sleep disruption and modified Ashworth scale (MAS) score.	[64]
**Synthetic** **Cannabidiol**	ZYN002 (transdermal synthetic cannabidiol gel)	Osteoarthritis	320	→ fulfil of American College of Rheumatology criteria for knee osteoarthritis; → 7 days wash-out of current anti-inflammatory and analgesic drugs, except paracetamol.	→ although ZYN002 was not statistically different from placebo, post-hoc analyses showed that men treated with ZYN002 had statistically significant reductions from baseline in average worst knee pain scores when compared with placebo group.	[65]
**Synthetic** **Δ9-tetrahydrocannabinol**	Nabilone	Alzheimer disease	38	→ patients with moderate to severe Alzheimer disease (AD); → age ≥55 years old; → fulfil of Diagnostic and Statistical Manual of Mental Disorders (DSM)-5 criteria for Major Neurocognitive Disorder due to AD, or met both Major Neurocognitive Disorder due to AD and Major Vascular Neurocognitive Disorder; → patients with a score of ≤24 on the standardized Mini-Mental Status Examination (sMMSE) and clinically significant agitation/aggression (Neuropsychiatric Inventory (NPI)-agitation/aggression subscore ≥3).	→ nabilone administration significantly reduced agitation over 6 weeks; → nabilone as associated with improvements on overall neuropsychiatric symptom; → nabilone showed greater improvements in agitation when compared to other cannabinoids; → nabilone was associated with significant → improvements on the short-form mini-nutritional assessment (MNA-SF), suggesting potential benefits on nutritional status.	[66]
**Synthetic** **Δ9-tetrahydrocannabinol**	Donabinol	Pancreatic cancer	104 estimated	→ patients ≥55 years od → locally advanced, inoperable or metastatic pancreatic cancer, eligible for first-line chemotherapy; → life expectancy of >4 months at screening; → female patients must either be post-menopausal or surgically sterilized or use a highly effective method of birth control (hormonal contraceptives, intra-uterine devices, or diaphragms with spermicide).	→ ongoing phase III clinical trial assessing the efficacy and safety of dronabinol in the improvement of chemotherapy-induced and tumor-related symptoms in advanced pancreatic cancer; → estimated completion date later in 2023 (NCT03984214).	

**Table 3:** Preclinical and clinical findings in cannabinoids use in Alzheimer’s disease.

Compound and Endocannabinoid System Targets	Experimental Model	Main Findings
**Cannabidiol** **mixed rCB1 and CB2 agonist**	→ treatment of PC12 cells with cannabidiol (10^−7^−10^−4^ m) prior to Aβ- peptide exposure	→ increased cell survival while it reduced lipid peroxidation, ROS production, caspase 3 levels, DNA fragmentation, and intracellular calcium [164]
	→ neuronal (SH-SY5Y) and microglial (BV-2) cell culture, 10 μM compound	→ neuroprotection against Aβ_1–42_ [165]
	→ primary mixed glial cells treated with 100 nM compound and 20 mg/kg of cannabidiol for 3 weeks delivered to Aβ-inoculated C57/Bl6 mice	→ reduce microglial activation and prevent Aβ-induced cognitive impairment and cytokine gene expression [151]
	→ 10 mg/kg of compound administered ip every other day for 2 weeks in 5xFAD mice	→ ameliorates cognitive function [166]
	→ daily administration of 0.75 mg/kg, ip compound for 5 weeks in AβPP/PS1 mice	→ improves cognitive performance, reduce Aβ deposition-related astrogliosis, and cytokine expression [167]
**ACEA** **rCB1 agonist**	→ a non-amnesic dose (1.5 mg/kg) of the compound for 5 weeks, administered ip to AβPP/PS1 transgenic mice	→ reduces the cognitive impairment [147]
**THC** **mixed rCB1 and CB2** **agonist**	→ N2a/AβPPswe cells treated with 100 μL THC	→ decrease Aβ levels [161]
	→ neuronal (SH-SY5Y) and microglial (BV-2) cell culture, 10 μM compound	→ neuroprotection against Aβ_1–42_ [165]
	→ THC was administered ip in 3 different doses (0.75, 1.5 and 3.0 mg/ kg) for 7 days (acute therapy) and 21 days (chronic treatment) in Spraque–Dawley rats	→ induce neurogenesis and improve cognitive performances of animals [168]
	→ 1.5 mg/kg/day of compound in ip delivery for 7 days in Sprague–Dawley rats	→ increases the expression, at both protein levels of BDNF and mRNA [27]
	→ daily administration of 0.75 mg/kg, ip compound for 5 weeks in AβPP/PS1 mice	→ reduces Aβ deposition-related astrogliosis and cytokine expression, improves cognitive performance [167]
	→ double-blind, randomized, placebo-controlled, crossover trial (10 old individuals with dementia), 12 weeks oral THC therapy (weeks 1–6, 0.75 mg; weeks 7–12, 1.5 mg)	→ encourages amyloidogenesis [169]
	→ an-open label 4 weeks pilot study in ten AD patients treated with different doses of THC (2.5 mg, 5 mg or 7.5 mg twice a day)	→ positive effects on mental state, dementia severity; and behavioral symptoms such as irritability, delusions, sleep [170]
	→ a double-blind study in mixed dementia and vascular patients and the administered dose was low (1.5 mg three times daily)	→ no improvement in neuropsychiatric symptoms score [171]
**WIN 55,212-2** **mixed rCB1 and CB2** **agonist**	→ 0.2 mg/kg/day of WIN 55,212-2 in the drinking water during 4 months in Tg APP 2576 mice	→ prevents neuroinflammation, increase Aβ clearance [158]
	→ human fetal astrocytes treated with 10 μM of compound for 72 h	→ inhibition of the production of inflammatory mediators (nitric oxide, cytokines, and chemokines) [172]
**HU210** **mixed rCB1 and CB2** **agonist**	→ 25 or 100 μg/kg of HU210 in ip administration for 10 days in Long-Evans, Wistar, and Fischer 344 rats	→ promote hippocampal neurogenesis and improves cognitive performance [173]
**JWH-133** **selective rCB2 agonist**	→ 0.2 mg/kg/day of JWH-133 in the drinking water during 4 months in Tg APP 2576 mice	→ improves cognitive performance and lowers β-amyloid levels [158]
	→ acute JWH133 injection of 0.2 mg/kg in Tg APP 2576 mice	→ enhances glucose uptake [174]

CB1 agonist ACEA, cannabinoid receptor type 1 (CB1) agonist arachidonyl-2-chloroethylamide (ACEA); ip, intraperitoneally; ROS, reactive oxygen species; 5xFAD mice, expressing human APP and PSEN1 transgenes with a total of five AD-linked mutations; THC, Δ 9-tetrahydrocannabinol; N2a/AβPPswe cells, N2a cells stably expressing 184 human AβPP carrying the K670N/M671L Swedish 185 mutation (APPswe); BDNF, brain-derived neurotrophic factor.

**Table 4:** The impact of cannabinoids receptor expression on cancer development and their implication on the disease prognosis.

Cancer Type	Receptor Expression	Effects	Ref.
**Colorectal cancer**	↑ CB2 ↑ CB1	↓ disease-free survival, ↓ overall survival, ↑ tumor growth ↓ disease outcome	[344,345]
**Prostate cancer**	↑ CB1	↑ Gleason score, ↑ incidence of metastases at diagnosis, ↑ tumor size, ↑ rate of proliferation, ↓ disease-specific survival	[340,341]
**Pancreatic cancer**	↓ CB1	↓ survival	[343]
**Head and neck squamos cell carcinoma**	↑ CB2	↓ survival	[346]
**Hepatocellular carcinoma**	↑ CB1 ↑ CB2	↑ disease-free survival	[337]
**Glioma**	↑ CB2	↑ tumor aggressivity	[338]
**Glioblastoma**	↑ CB2	↑ histologic grade	[347]
**Non-small cell lung cancer**	↑ CB1, ↑ CB2	↑ survival	[339]

↑ = increase of; ↓ = decrease of.

## Data Availability

Not applicable.
